# Generation and characterization of inducible KRAB-dCas9 iPSCs from primates for cross-species CRISPRi

**DOI:** 10.1016/j.isci.2024.110090

**Published:** 2024-05-23

**Authors:** Fiona C. Edenhofer, Anita Térmeg, Mari Ohnuki, Jessica Jocher, Zane Kliesmete, Eva Briem, Ines Hellmann, Wolfgang Enard

**Affiliations:** 1 Anthropology and Human Genomics, Faculty of Biology, Ludwig-Maximilians-Universität München, 82152 Planegg, Germany; 2 Institute for the Advanced Study of Human Biology, Kyoto University, Kyoto 606-8501, Japan; 3 Hakubi Center, Kyoto University, Kyoto 606-8501, Japan

**Keywords:** molecular biology

## Abstract

Comparisons of molecular phenotypes across primates provide unique information to understand human biology and evolution, and single-cell RNA-seq CRISPR interference (CRISPRi) screens are a powerful approach to analyze them. Here, we generate and validate three human, three gorilla, and two cynomolgus iPS cell lines that carry a dox-inducible KRAB-dCas9 construct at the AAVS1 locus. We show that despite variable expression levels of KRAB-dCas9 among lines, comparable downregulation of target genes and comparable phenotypic effects are observed in a single-cell RNA-seq CRISPRi screen. Hence, we provide valuable resources for performing and further extending CRISPRi in human and non-human primates.

## Highlights

• Generation and validation of eight primate iPSC lines for dox-inducible CRISPRi
• Comparable knockdowns despite variable KRAB-dCas9 levels among cells
• Quantitative association between SOX2 knockdown and transcriptome profile
• Single-cell CRISPRi screen for 24 transcription factors in human and cynomolgus

## Graphical abstract

**Figure:**
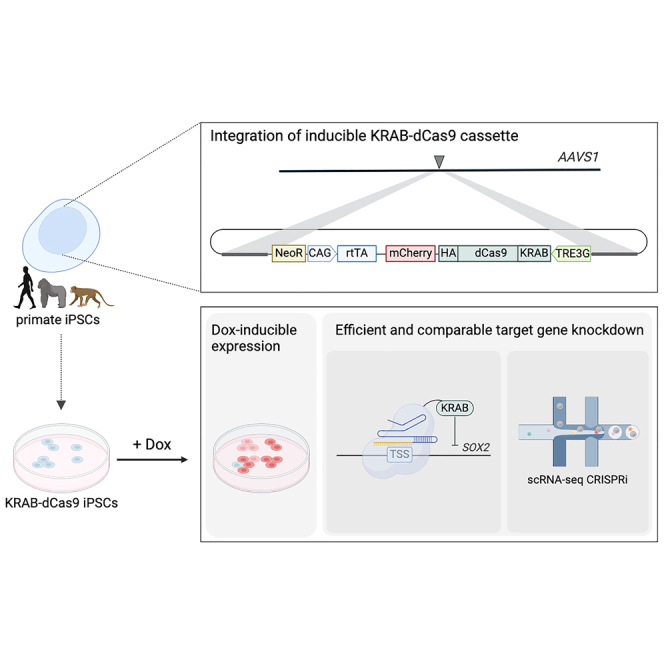

## Introduction

Cross-species comparisons are essential to understand human biology, disease, and evolution.^1^^,^^2^ On the DNA level, comparative genomics enables increasingly fine-grained estimates of constraint across vertebrates,^3^ mammals,^4^ and primates^5^ and starts to reveal evolutionary genotype-phenotype associations, especially when combined with epigenetic data^6^ or functional assays.^7^ On the level of molecular phenotypes, comparisons of chromatin states, transcription factor binding or expression levels have revealed patterns and mechanisms of gene regulatory evolution.^8^^,^^9^^,^^10^^,^^11^^,^^12^ Furthermore, correlations with higher level phenotypes could be discovered, such as human-specific properties of brain development.^13^^,^^14^^,^^15^^,^^16^ However, the potential of phenotypic comparisons across species - especially for developmental processes - is still impeded by limited access to homologous and experimentally accessible cell types from sufficient individuals and species.^17^^,^^18^ Induced pluripotent stem cells (iPSCs) can enable such an access and an increasing amount of iPSCs from different non-human primates have been generated in recent years.^19^^,^^20^^,^^21^ Genetically accessible iPSCs from primates can then be combined with CRISPR screens to efficiently study the evolution of genotype-phenotype relationships.

In CRISPR screens, a nuclease like Cas9 is targeted by guide RNAs (gRNAs) to edit, activate, or silence several target genes.^22^^,^^23^ One powerful variant is CRISPR interference (CRISPRi), in which a catalytically deactivated Cas9 (dCas9) is fused to a repressor domain like the Krüppel associated box (KRAB) protein. When targeted by a gRNA to a promoter, this leads to epigenetic repression and consequently to a knockdown of the promoter-associated gene.^24^^,^^25^^,^^26^ Such knockdown screens can be advantageous in comparison to Cas9 knockout, as they are reversible, more homogenous and induce less DNA damage-associated toxicity.^27^^,^^28^^,^^29^ Using a CRISPRi screen with gRNA counting as readout, recently revealed unexpected differences in gene dependencies affecting cell cycle control and cell proliferation between human and chimpanzee, exemplifying the relevance of primate comparisons to understand human evolution.^30^ CRISPR screens that use gRNA abundance as readout are efficient for studying mechanisms that influence phenotypes like cell survival or proliferation, but high-content readouts such as single-cell RNA-sequencing (scRNA-seq) offer even more complex phenotypic information.^22^ While numerous remarkable single-cell CRISPR screens have been performed in individual species,^31^^,^^32^^,^^33^^,^^34^^,^^35^^,^^36^ cross-species approaches would provide unique additional and functionally relevant information. In particular, comparative single-cell CRISPR screens that would be conducted across different primate species, would allow us to compare perturbation effects among primates on a single-cell level and with that decipher the evolution of genes and gene regulatory networks.

Key to such a comparative approach is the comparability of cells from different species. In addition to the necessary biological replicates,^18^ this includes a strategy to isolate comparable and efficient dCas9-expressing clones e.g., for a CRISPRi screen. One starting point to increase comparability among clones is the directed integration of the dCas9-encoding construct into a specific locus, like the adeno-associated virus integration site 1 (AAVS1) safe-harbor locus, to avoid variation due to different insertion sites, disruption of cellular genes, and to prevent transgene silencing.^37^^,^^38^

While the AAVS1 locus has been well established in human cells and has also been used as a target site in some primates,^30^^,^^39^^,^^40^ in this study, we show the utility of transgene integration at the AAVS1 locus in additional non-human primate iPSCs. More precisely, we present the generation and characterization of inducible KRAB-dCas9 iPSCs of the three primate species human (*Homo sapiens*), gorilla (*Gorilla gorilla*), and cynomolgus (*Macaca fascicularis*). To this end, we constructed species-specific donor plasmids harboring a ZIM3-KRAB-dCas9-HA-P2A-mCherry coding cassette (from here on called KRAB-dCas9 cassette). We show integration of the construct into the AAVS1 locus of the targeted species while ensuring the pluripotent state of the knock-in iPSCs. We demonstrate inducible expression of the KRAB-dCas9 cassette in the generated human, gorilla, and cynomolgus KRAB-dCas9 iPSCs, and despite varying KRAB-dCas9 expression levels, we observe comparable gene knockdown efficiencies among species. In summary, we provide an important resource and methodology to leverage CRISPR screens for cross-primate comparisons.

## Results

### Generation of inducible, AAVS1-targeted KRAB-dCas9 primate iPSCs

**Figure 1:**
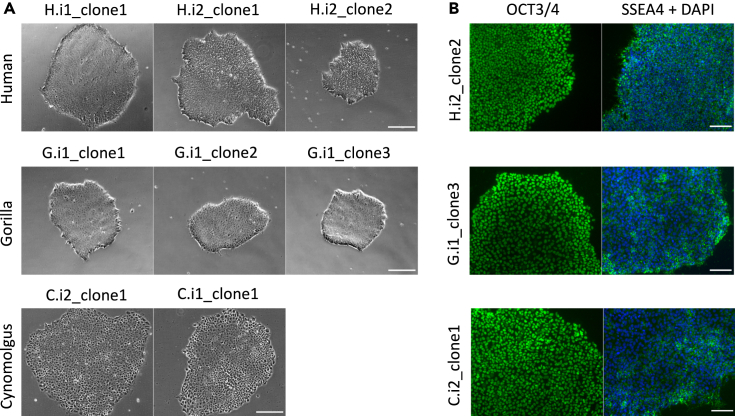
Generation of inducible, AAVS1-targeted KRAB-dCas9 primate iPSCs (A) The generated KRAB-dCas9 iPS cell-lines show classical iPSC colony morphology characterized by tightly packed cells and clearly defined colony edges; scale bar represents 250 μm. (B) Immunofluorescence stainings of the pluripotency markers OCT3/4 and SSEA4. Here shown for human H.i2_clone2, gorilla G.i1_clone3, and cynomolgus C.i2_clone1 (see also Figure S2); scale bar represents 100 μm.

For the generation of the KRAB-dCas9 iPSCs, human, gorilla, and cynomolgus wild-type (wt) iPSCs were utilized, which were previously generated and validated by our group^17^^,^^41^(see method details). Knock-in at the AAVS1 locus was performed with ZnF nucleases targeting the human and gorilla AAVS1 locus, or with eCas9 targeting the cynomolgus AAVS1 locus. The KRAB-dCas9 cassette, encoded on the donor plasmid, was stably integrated into the AAVS1 locus of the primates. To maximize knockdown efficiency, we used dCas9 fused to the ZIM3 KRAB domain, which has recently been found to be the most efficient repressor domain.^26^^,^^29^ The expression of the KRAB-dCas9 cassette is controlled by the Tet-On system consisting of constitutively active transactivator expression and tet-operators upstream of KRAB-dCas9 that can be induced by addition of doxycycline (dox),^42^ allowing time-controlled perturbation. The construct is flanked by species-specific AAVS1-targeting homology arms, which enable integration into the AAVS1 locus via homologous recombination^37^ and can be exchanged in one cloning step, allowing for integration of the same KRAB-dCas9 cassette into the genome of different species (see method details). The generated KRAB-dCas9 iPSC clones were expanded and integration of the construct was confirmed by genotyping PCRs, also revealing homozygous integration for clone H.i2_clone2 (Figures S1A and S1B). After knock-in, a typical iPSC-like colony morphology, characterized by tightly packed cells, and clearly defined colony edges^43^ could be observed for all KRAB-dCas9 iPS cell lines of all species (Figure 1A). Furthermore, all generated knock-in clones showed a high expression of OCT3/4 and SSEA4, indicating that pluripotency of the parent lines could be preserved (Figures 1B and S2).

### Induced expression levels of KRAB-dCas9 are variable among clones

**Figure 2:**
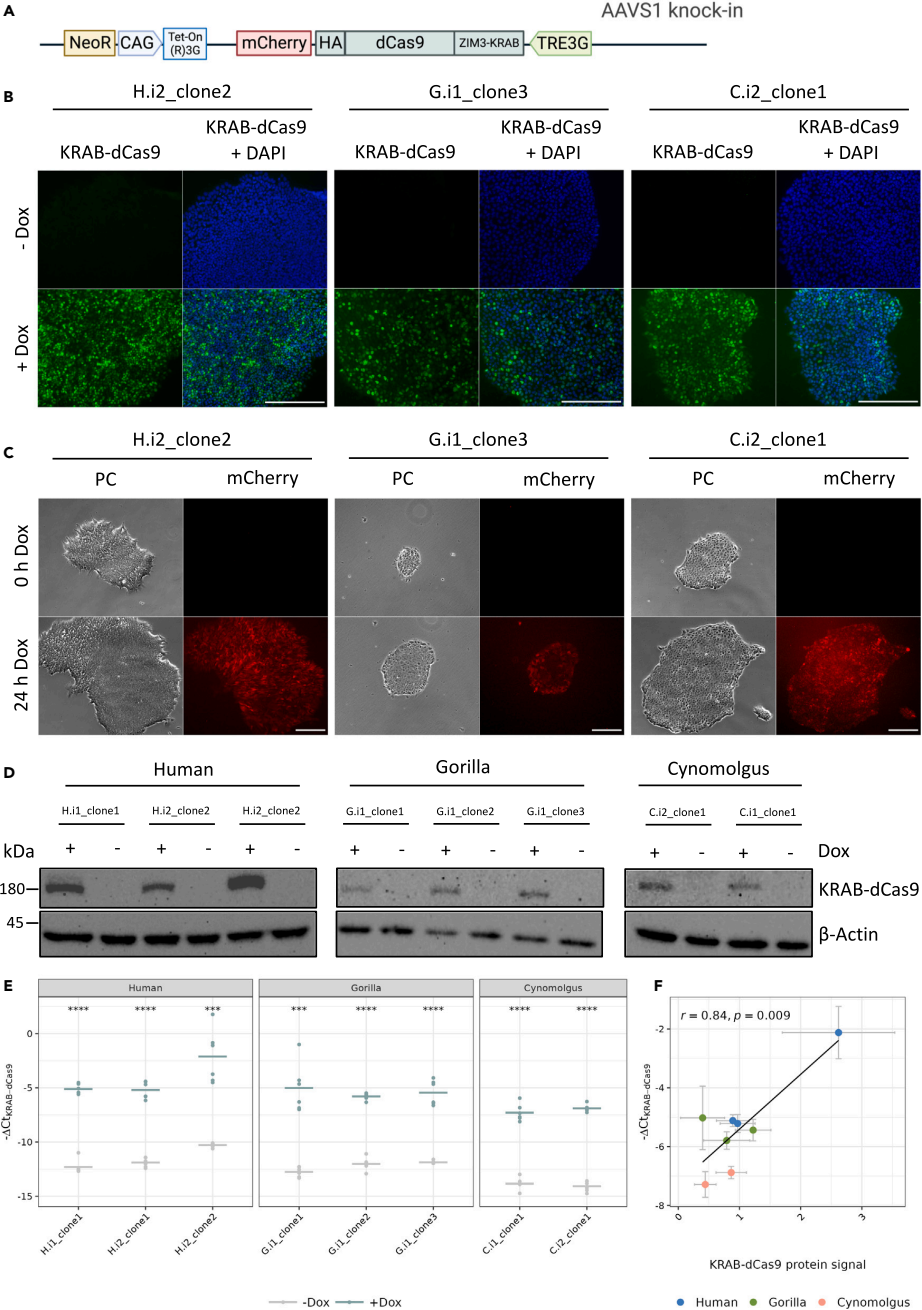
Induced expression levels of KRAB-dCas9 are variable among clones (A) Schematic representation of the AAVS1 locus after knock-in of the KRAB-dCas9 cassette (NeoR = neomycin resistance gene, CAG = chicken beta actin promoter, Tet-On(R)3G = transactivator protein, TRE3G = tetracycline response element, consisting of 7 repeats of a 19 nucleotide tetracycline operator sequence). (B) Immunofluorescence stainings for KRAB-dCas9 (via detection of the fused HA-tag) of human H.i2_clone2, gorilla G.i1_clone3, and cynomolgus C.i2_clone1 (see also Figure S3). Before fixation, KRAB-dCas9 iPSCs were cultured in medium with or without 1 μg/mL dox for 4 days; scale bar represents 250 μm. (C) Phase contrast and mCherry-signal images of an iPSC colony after 0 h and after 24 h of dox-treatment for human H.i2_clone2, gorilla G.i1_clone3, and cynomolgus C.i2_clone1 (mCherry-signal was normalized within each species and signal intensities are not representative as comparison between the species); scale bar represents 250 μm. (D) Western blot analysis for KRAB-dCas9 (via detection of the fused HA-tag) and beta-actin of human, gorilla, and cynomolgus KRAB-dCas9 iPSC clones that were cultured in medium with or without 1 μg/mL dox for 4 days. (E) qPCR analysis of KRAB-dCas9 expression. y axis shows negative ΔCt values of KRAB-dCas9 compared to GAPDH, i.e., normalized KRAB-dCas9 expression on a log2 scale. Cells were cultured in medium with or without 1 μg/mL dox for 4 days, before total RNA extraction. Horizontal lines indicate the mean (*n* = 6); Results of paired t tests indicate significant differences between the mean of the KRAB-dCas9 ΔCt in the +dox and the ˗dox condition (0 '∗∗∗∗' 0.0001 '∗∗∗' 0.001 '∗∗' 0.01 '∗' 0.05 'ns' Inf). (F) Significant correlation of KRAB-dCas9 negative ΔCt and KRAB-dCas9 protein signal that was quantified from western blots (*n* ≥ 2). Data are represented as mean ± SEM; Pearson’s *r* = 0.84, *p* -value = 0.009.

One main advantage of the integrated construct is the inducibility of KRAB-dCas9 expression. A time-controlled start of KRAB-dCas9 expression and subsequent gene knockdown can be essential to differentiate between early/primary and late/downstream effects of a perturbation.^44^ Furthermore, to study perturbation effects during differentiation, a precisely controlled start of nuclease expression can be advantageous.^45^ In our construct, the transactivator protein Tet-On(R)3G is constantly expressed under control of a chicken beta-actin (CAG) promoter, while expression of KRAB-dCas9-HA-mCherry is under control of a dox-inducible TRE3G promoter (Figure 2A). Furthermore, the opposite coding direction of the Tet-On(R)3G coding sequence should avoid leaky expression of KRAB-dCas9. To test inducibility, KRAB-dCas9 iPSCs were cultured for 4 days in medium with or without dox and then fixed and stained for the HA-tag which is fused to the C-terminus of dCas9 and hence, representative for KRAB-dCas9 expression (Figure 2A). All clones of all species were clearly KRAB-dCas9-negative when not treated with dox and clearly KRAB-dCas9-positive when treated with dox, albeit with different intensities among cells of a clone (Figures 2B and S3).

In our construct mCherry is linked to the C-terminus of KRAB-dCas9 via a 2A self-cleaving peptide (P2A), allowing us to observe the expression in living cells over time at a cellular resolution (Figure 2A). The same colony of a clone was compared at 0 h and 24 h after culturing in dox-containing medium. While no signal was present at 0 h, after 24 h an mCherry-signal could be observed in all colonies. Here, we noted different signal intensities within a colony, indicating a heterogeneous expression among cells of one colony (Figure 2C). To enrich cells with a high expression, we sorted cells of three clones (H.i1_clone1, C.i1_clone1, C.i2_clone1) for a high mCherry-signal by flow cytometry. However, similar expression heterogeneity was restored already after one passage, indicating that expression levels are not heritable over a few cell divisions (Figures S1C and S1D).

Next, we analyzed KRAB-dCas9 protein levels of different clones by western blotting. We cultured cells with and without dox as described previously and detected a band at the expected molecular weight only in dox-treated cells (Figure 2D), again confirming the inducibility of protein expression by dox. Quantification of the intensity of the detected bands revealed different expression levels, indicating variable KRAB-dCas9 expression not only within but also among clones. To quantify this further, qPCR was performed which showed a significant 74– to 288-fold increase of KRAB-dCas9 expression levels (normalized to GAPDH expression levels) upon dox treatment (Figure 2E). Furthermore, it also revealed different expression levels among clones that correlated well with the protein levels among clones (Figure 2F). In summary, we find that expression of KRAB-dCas9 is dox-inducible in all clones, at different levels among clones and also variable among cells of the same clone. Next, we tested to what extent this variation might affect the knockdown efficiency.

### Efficient *SOX2* knockdown can be induced in the KRAB-dCas9 iPSCs

**Figure 3:**
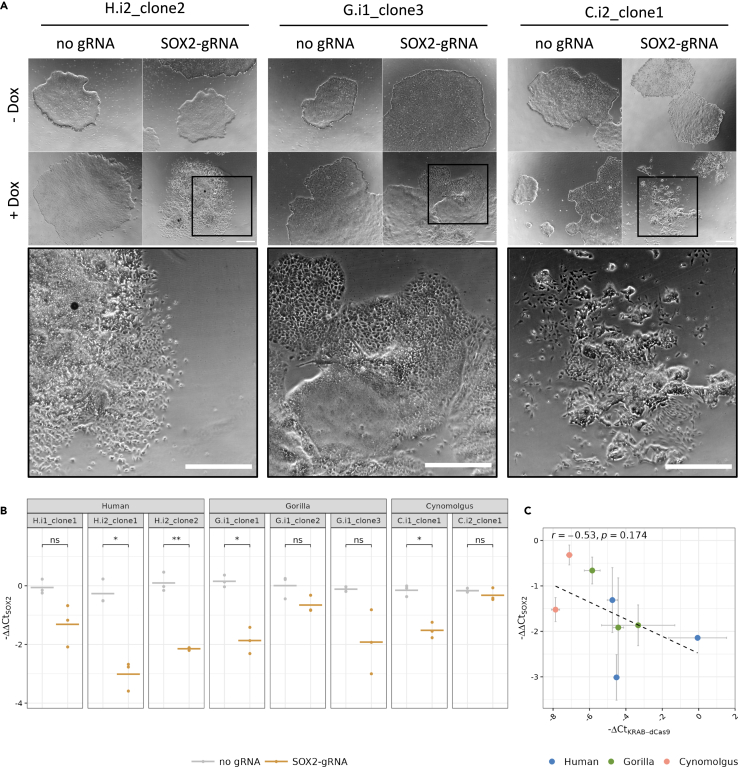
Efficient *SOX2* knockdown can be induced in the KRAB-dCas9 iPSCs (A) Phase contrast images of human H.i2_clone2, gorilla G.i1_clone3, and cynomolgus C.i2_clone1. KRAB-dCas9 iPSCs were either transduced with a *SOX2*-targeting gRNA or remained non-transduced and were cultured in medium with or without 1 μg/mL dox for 4 days, respectively. Close-up of the cells that were transduced with a *SOX2*-targeting gRNA and cultured under dox-conditions; scale bar represents 500 μm. (B) SOX2 transcripts of perturbed or unperturbed cells were quantified by qPCR for the three human, three gorilla and two cynomolgus KRAB-dCas9 iPS cell lines. Expression levels were normalized to GAPDH and relative *SOX2* transcript expression between dox-treated and untreated cells was determined using the ΔΔCt method. Negative ΔΔCt-values comparing the +dox and ˗dox condition are shown for *SOX2*. Horizontal lines indicate the mean; (*n* = 3 technical replicates). Results of paired t tests indicate differences between the mean of the samples with and without a *SOX2*-targeting gRNA (0 '∗∗∗∗' 0.0001 '∗∗∗' 0.001 '∗∗' 0.01 '∗' 0.05 'ns' Inf). A t test across species between all samples with and all samples without a *SOX2*-targeting gRNA revealed a significant difference of the SOX2 ΔΔCt (*p* value = 4.5 x 10^−8^). (C) Negative ΔΔCt of SOX2 in perturbed cells compared to negative ΔCt of KRAB-dCas9. Data are represented as mean ± SD; Pearson’s *r* = −0.53, *p* value = 0.174.

To assess the functionality of the KRAB-dCas9 iPSCs for CRIPSRi, we performed a *SOX2*-perturbation assay, as downregulation of *SOX2* is known to induce loss of pluripotency which can be detected as differentiation of iPSCs.^46^ Human, gorilla, and cynomolgus KRAB-dCas9 iPSCs were transduced with a *SOX2*-targeting gRNA cloned into the CROP-seq-opti vector,^47^ selected for vector integration by puromycin, and then cultured with or without dox for 4 days. For SOX2-gRNA-transduced cells that were cultured in dox-containing medium, we observed a clear loss of colony borders and an increased number of differentiated cells 4 days after dox addition, indicating loss of pluripotency of those cells. In contrast, no changes of iPSC colony morphology were observed in transduced cells without dox or in non-transduced cells independent of the dox condition (Figure 3A).

In an independent experiment, we quantified *SOX2* mRNA levels by qPCR. In cells transduced with a SOX2-gRNA, *SOX2* mRNA levels ranged between 0.8-fold (20% knockdown) and 0.12-fold (88% knockdown) (average 0.38-fold, 62% knockdown) in cells treated with dox, compared to cells of the same clone not treated with dox. As expected, in non-transduced cells the *SOX2* mRNA levels did not change upon dox treatment (Figure 3B).

Further, we found that the magnitude of *SOX2* knockdown did not correlate significantly with the mRNA levels of KRAB-dCas9 (Pearson’s *r* = −0.53; *p* value = 0.17; Figure 3C). For example, an ∼8-fold difference in KRAB-dCas9 levels between clones resulted in a ∼4-fold *SOX2* reduction in both clones and similar KRAB-dCas9 levels resulted in ∼2-fold, 4-fold, or 8-fold *SOX2* reduction (Figure 3C). Hence, KRAB-dCas9 levels do not seem to be the limiting factor for CRISPRi knockdown of *SOX2*.

**Figure 4:**
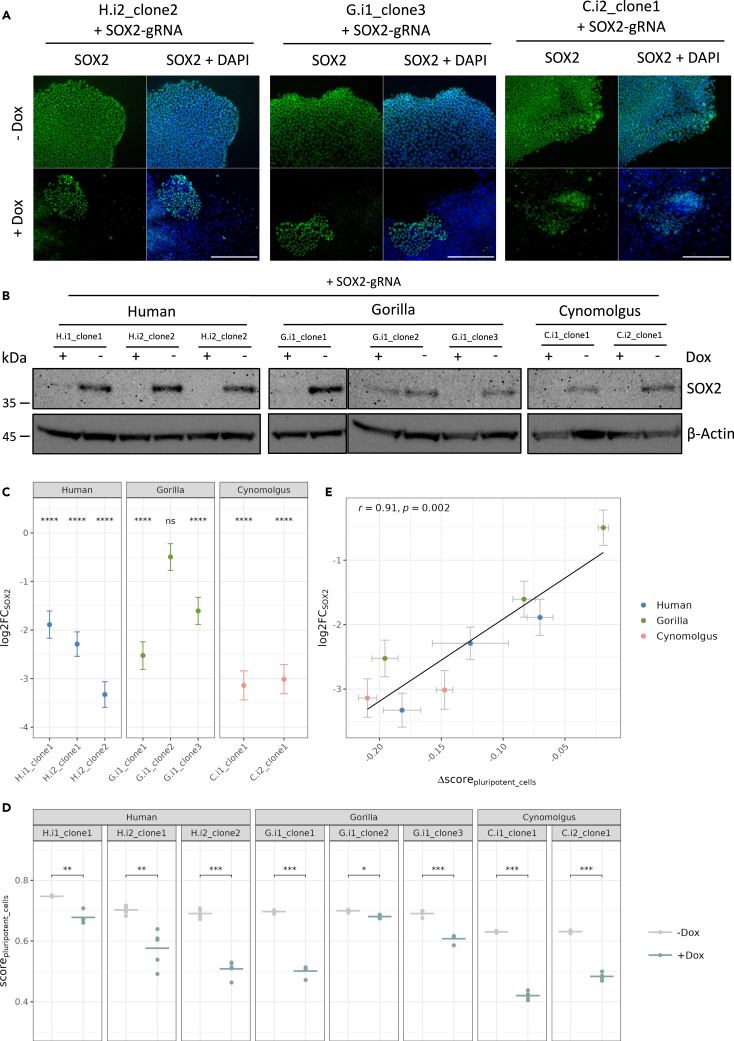
SOX2 knockdown in the KRAB-dCas9 iPSCs leads to reduced association with a pluripotent cell profile (A) Immunofluorescence stainings of SOX2 in human H.i2_clone2, gorilla G.i1_clone3, and cynomolgus C.i2_clone1 cells. KRAB-dCas9 iPSCs with an integrated SOX2-targeting gRNA were cultured in medium with or without 1 μg/mL dox for 4 days; scale bars indicate 250 μm. (B) Western blot analysis for SOX2 and beta-actin of human, gorilla, and cynomolgus KRAB-dCas9 iPSC clones with an integrated SOX2-targeting gRNA. Cells were cultured in medium with or without 1 μg/mL dox for 4 days, before protein extraction. (C) log2FC of the *SOX2* expression level in KRAB-dCas9 iPSCs with an integrated *SOX2*-targeting gRNA between +dox and ˗dox condition; error bars indicate SEM; (∗∗∗∗ (*p*.adj ≤ 0.0001), ns (*p*.adj > 0.05)). (D) Cell type classification was performed using SingleR^49^ with reference data from Rhodes et al.^50^ (see also Figure S4). Correlation scores of the samples to pluripotent cells of the reference data are shown for the +dox and ˗dox condition; results of paired t tests indicate significant differences of the mean scores between the two conditions (0 '∗∗∗∗' 0.0001 '∗∗∗' 0.001 '∗∗' 0.01 '∗' 0.05 'ns' Inf); note that the reference data were generated from human samples only and hence lower correlation scores are expected for more diverged primates. (E) Correlation of the Δscore for pluripotent cells between the +dox and the ˗dox score to the log2FC of *SOX2* expression; data are represented as mean (n = 4–5 biological replicates) +/− SEM; Pearson’s *r* = 0.91, *p* value = 0.002.

In addition to the analysis of *SOX2* transcript levels, we also investigated the knockdown of the SOX2 protein. In immunofluorescence stainings, we found the SOX2 signal to be clearly reduced in the +dox (*SOX2* knockdown) condition, as compared to the ˗dox condition, with still some SOX2-positive cells per colony (Figures 4A and S4A). In Western Blots, the SOX2 signal was also clearly reduced in the +dox condition, as compared to the ˗dox condition (Figure 4B). This reduction was below the detection limit except for the G.i1_clone2 that also showed a relatively weak reduction on the RNA level before. In summary, CRISPRi of SOX2 did work in our generated clones with variable magnitudes of repression. This variability did not depend much on KRAB-dCas9 levels—at least not for the range of levels present in our clones. A negative aspect of this finding is that similar KRAB-dCas9 levels are no strong criterion for comparability among clones, but a positive aspect is that different levels are no strong exclusion criterion, either.

### *SOX2* knockdown in the KRAB-dCas9 iPSCs leads to reduced association with a pluripotent cell profile

To analyze the effects of the *SOX2* knockdown, we cultured the eight populations of KRAB-dCas9 iPSCs carrying the *SOX2*-targeting gRNA for four days with and without dox in four to five biological replicates each. Bulk RNA-seq of the resulting 68 samples was performed using prime-seq.^48^ Quantifying SOX2 levels revealed different degrees of SOX2 repression (Figure 4C), in agreement with the qPCR and western blot analyses described previously. As already indicated by a change in colony morphology of the *SOX2*-knockdown cells (Figure 3A), we expected that transcription profiles would change from pluripotent profiles to profiles of more differentiated cells. To quantify this, we used SingleR^49^ to calculate correlation scores to expression profiles of a published dataset.^50^ As expected, all replicates of all eight clones showed the highest correlation with pluripotent cells under ˗dox conditions (Figure S4C). This score was significantly reduced under +dox conditions for all clones (Figure 4D). Correlation scores for differentiated cells, such as early ectoderm, neural crest, endoderm, and especially mesoderm tended to increase in the +dox as compared to the ˗dox condition (Figure S4B). However, it did not show a clear trend toward a particular lineage, as an assignment toward a lineage using the highest correlation score was quite variable among clones and replicates under +dox conditions (Figure S4C). In summary, *SOX2* knockdown consistently led to a loss of pluripotency in all clones. Remarkably, the magnitude of *SOX2* knockdown correlated strongly with the magnitude of the decrease of the correlation score with pluripotent cells (Pearson’s *r* = 0.91, *p* value = 0.002; Figure 4E), indicating that similar levels of CRISPRi repression, at least in the case of *SOX2*, lead to comparable downstream effects across clones.

### Knockdown efficiency is comparable in human and cynomolgus KRAB-dCas9 iPSCs in a single-cell CRISPRi screen

**Figure 5:**
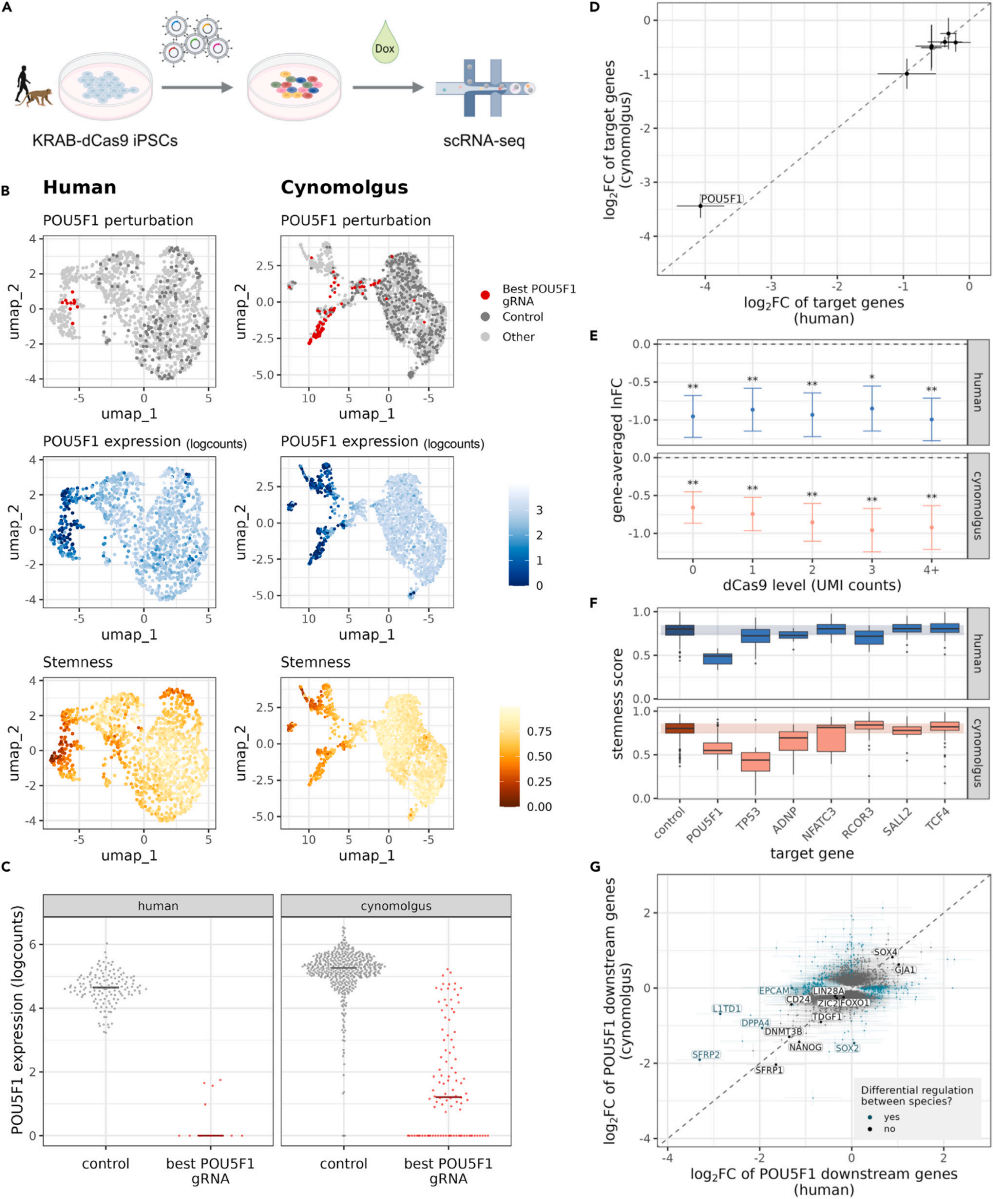
Knockdown efficiency is comparable in human and cynomolgus KRAB-dCas9 iPSCs in a single-cell CRISPRi screen (A) Workflow of the single-cell CRISPRi screen targeting 24 transcription factor genes in human and cynomolgus KRAB-dCas9 iPSCs. After transduction with species-specific gRNA libraries, the successfully transduced cells were cultured for 5 days with doxycycline to induce KRAB-dCas9 expression, before performing single-cell RNA-seq. (B) UMAP embeddings of the scRNA-seq data from the CRISPRi screen. From top to bottom, cells are colored by *POU5F1* perturbation, with the most effective *POU5F1*-targeting gRNA highlighted in red, *POU5F1* expression in logcounts, and stemness score based on a one-class logistic regression model trained on the PCBC (Progenitor Cell Biology Consortium) dataset.^71^ (C) *POU5F1* was successfully downregulated in human and cynomolgus cells. Data points show *POU5F1* expression in control cells with non-targeting gRNAs and in perturbed cells with the best *POU5F1*-targeting gRNA in each species. Median expression levels are marked. (D) Target genes are downregulated to a similar degree in human and cynomolgus cells. We plot log2FCs^72^ with 95% confidence intervals for the best performing gRNA for the 7 target genes that were significantly downregulated and fulfilled minimal detection criteria in both species. (E) Target gene-averaged ln fold changes between perturbed and control cells as estimated using a linear mixed model to predict the target gene expression with discretized KRAB-dCas9 expression levels (0/1/2/3/4+ UMIs/cell) as fixed effect and random effects for target gene nested with gRNA identity (see STAR Methods for details). All lnFC estimates are negative and significant, showing that the targets are downregulated in case of all KRAB-dCas9 levels (significance codes based on Tukey’s HSD test: 0 '∗∗∗' 0.001 '∗∗' 0.01 '∗' 0.05 '.' 0.1 ' ' 1; error bars represent standard errors). (F) Stemness scores for the control cells and the 7 target genes in D. The cells with POU5F1, TP53, and ADNP knockdown have significantly lower stemness than the control cells in both human and cynomolgus. (G) Downstream effects of the POU5F1 perturbation on the transcriptome. The log2 fold changes (perturbed VS control) in cynomolgus are plotted against the log2 fold changes in human for the genes that are significantly differentially expressed in at least one species. Data points are colored by differential regulation between species (significance threshold for differential expression and differential regulation: adj. *p* value < 0.05). 15 known pluripotency markers and *POU5F1* targets are labeled; they are all highly differentially expressed in human, cynomolgus or both. 5 of them were found to be differentially regulated between the species, while for 10 of them we detected no significant species difference.

After confirming the inducible expression of KRAB-dCas9 and the efficient knockdown of *SOX2* in the generated KRAB-dCas9 iPSCs, we tested the applicability and comparability of the cell lines in a cross-species perturbation setup and performed a single-cell CRISPRi screen (Figure 5A). We limited this proof-of-principle experiment to two species to optimize costs and experimental complexity and picked human and cynomolgus to cover the longer and more challenging phylogenetic distance among our clones. To demultiplex clones by SNPs in one single-cell experiment, we needed clones from different individuals, and we wanted to pick clones that had shown an efficient *SOX2*-knockdown in all previous experiments. This led to the choice of H.i1_clone1, H.i2_clone2, and C.i1_clone1. We targeted 24 transcription factors in human and cynomolgus with 4 gRNAs each. We cloned these gRNAs as well as 15 non-targeting gRNAs into the CROP-seq-opti vector, to create two separate species-specific gRNA libraries. Human and cynomolgus KRAB-dCas9 iPSCs (H.i1_clone1, H.i2_clone2, and C.i1_clone1) were transduced with the lentiviral pool of gRNAs with a MOI of 0.1. Transduced cells were selected with puromycin and then cultured in dox-containing medium for 5 days, before they were harvested for scRNA-seq. Using the 10X Genomics platform, we generated 5′ gene-expression libraries with additional gRNA libraries to improve gRNA capture in the cells. We recovered ∼7,700 cells in total and after QC and demultiplexing, had 2,855 human and 3,053 cynomolgus cells with a median unique molecular identifier (UMI) count of 17,870 and 16,114 per cell, respectively. After removing cells with low gRNA expression or with several different gRNAs above noise level, 90.2% of the human and 78.8% of the cynomolgus cells were associated with a single dominant gRNA.

We first focused on cells that were associated with *POU5F1* (*OCT4*)-gRNAs, given its prominent role in pluripotency.^51^ UMAP embeddings showed that cells containing the best *POU5F1*-targeting gRNA cluster together in both species (Figure 5B). Furthermore, the *POU5F1*-gRNA clusters were associated with decreased *POU5F1* expression as well as reduced stemness gene expression signature (Figures 5B and 5C). Hence, similar to *SOX2* aforementioned, we observe expected effects upon knockdown of a gene known to maintain a pluripotent state.

Generally, we found most target genes to be significantly downregulated (71% median knockdown in human cells, max. 96%; 59% median knockdown in cynomolgus cells, max. 94%; Figure S5A). Most importantly for our comparative approach, when taking the best gRNAs for each gene in each species, we found that downregulation of the targets happened to a similar degree in human and cynomolgus (Figure 5D). In order to quantify the effect of KRAB-dCas9 expression levels on target gene downregulation, we fitted a linear mixed effects model with KRAB-dCas9 levels (0/1/2/3/4+ UMIs/cell) as main predictor, while controlling for target genes and gRNAs. We found the resulting gene averaged log fold change estimates to be negative and significant, proving a downregulation effect for all KRAB-dCas9 levels (Figure 5E). Even for perturbed cells where no KRAB-dCas9 transcript was detected (level 0), the estimates indicated significant downregulation effects, suggesting that low levels of KRAB-dCas9 can be sufficient (see also Figures S5B and S5C).

When comparing target expression among perturbed cells with different KRAB-dCas9 levels, a weak negative trend toward higher levels is visible for the cynomolgus (Figure 5E), similar to what was observed in the qPCR of *SOX2* (Figure 3C). However, none of the contrasts between the KRAB-dCas9 levels were found to be significant. In summary, these analyses show the efficient and also comparable knockdown of target genes in the generated KRAB-dCas9 iPSCs, largely independent of differences in KRAB-dCas9 level that exist among cells of the same clone or among populations of cells of different clones.

Next, we evaluated the downstream effects of the perturbations on cell fate and gene expression. When comparing the stemness scores of perturbed and control cells, we found that the knockdown of several transcription factors, including the pluripotency factor *POU5F1*, led to a significant decrease in stemness both in human and cynomolgus iPSCs (Figure 5F), similar to the effect of the *SOX2* knockdown in the bulk RNA-seq experiment.

We also identified downstream genes significantly repressed or activated upon each perturbation and tested whether the effects on these genes are different between the two species (Figures 5G and S5D; Table S4). In case of *POU5F1*, the perturbation altered the expression of 2,445 downstream genes in total, out of which 495 were significantly differentially regulated between the two species and 1,950 were not. The affected genes included well-established pluripotency markers (*NANOG*,^52^^,^^53^ *SOX2*,^52^ *DNMT3B*,^54^ *L1TD1*,^55^ *EPCAM*,^56^ *LIN28A*,^53^^,^^55^ *CD24*,^57^^,^^58^ *ZIC2*,^58^^,^^59^ SFRP1,^60^ and SFRP2^53^^,^^58^), as well as known *POU5F1* targets (*NANOG*,^61^ *SOX2*,^61^ *DPPA4*,^61^ *TDGF1*,^61^ *FOXO1*,^61^ *GJA1*,^61^ and *SOX4*^62^) identified in ChIP-on-chip and ChIP-seq studies (Figure 5G). Five of these highlighted genes, including *SFRP2*, *L1TD1*, and *SOX2*, were found to be differentially regulated between human and cynomolgus in our screen.

These results confirm that the perturbations have a detectable effect on the gene regulatory networks of the transcription factors and often also on cell fate. For example, the perturbation of *POU5F1* led to the downregulation of many known pluripotency markers and *POU5F1* targets and promoted an exit from the stem cell-like state, in line with our expectations. Future larger-scale perturbation screens using our primate KRAB-dCas9 iPS cell lines can help infer novel gene regulatory links and investigate transcriptional network rewiring across species.

## Discussion

Comparisons of molecular and cellular phenotypes across primates leverage unique information to understand human biology and evolution. Powerful methodologies such as generation of iPSCs in combination with functional genomic readouts have added exciting possibilities to this approach in recent years,^2^^,^^16^^,^^19^ including the possibility to perform genome-wide gene perturbations by CRISPR screens.^30^ Key to such an approach is generating comparable, i.e., homologous cell types across a sufficient number of biological replicates per species.^16^^,^^18^ However, iPSCs are known to differ (epi)genetically among individuals, among iPSC clones of the same individual and among cells within a clone. Hence e.g., pro-survival adaptations such as deletions in p53^63^ can be easily mistaken as species differences.^30^ As genetic modification and clonal selection are necessary to generate stable, nuclease-expressing cells for CRISPR screens, the knock-in process could add additional, unwanted variation that one needs to control for.^64^ While isogenic iPSCs that share a single genetic background are a potential solution to control for much of this unwanted variation, this is obviously not possible when working with different species. Notably, generating isogenic iPSCs is also no option when studying patient-derived iPSCs for polygenic diseases. Hence, our results on generating comparable, CRISPRi-suitable iPSCs from different primates are also relevant when generating comparable iPSCs from patients and controls.

We present the generation and validation of comparable KRAB-dCas9 iPSC lines of three human, three gorilla, and two cynomolgus monkey clones that are suitable for CRISPRi. To avoid variation due to random integration of the dox-inducible KRAB-dCas9 construct, we inserted the KRAB-dCas9 cassette at the established safe-harbor locus AAVS1. We performed multiple verifications and a *SOX2*-knockdown experiment for all clones to verify and compare transgene expression and to test target gene knockdown efficiency, before using two of the clones that consistently showed good results in a cross-species single-cell CRISPRi screen. Here, we could confirm the expected dox-inducible expression of the KRAB-dCas9 cassette on an RNA and protein level but observed substantial variation in expression levels of KRAB-dCas9 among and within the generated lines. To assess to what extent this variation impairs the comparability of gene perturbation of the cell lines, we compared *SOX2* downregulation and its cellular consequences for all generated clones. We confirmed *SOX2*-knockdown on an RNA and protein level and could link different levels of knockdown to the extent of subsequent cell fate changes. Lastly, we compared downregulation of 24 transcription factors in a human and cynomolgus line in a cross-species CRISPR screen, using scRNA-seq as a readout. We found that we can achieve significant and comparable downregulation of targets despite variable levels of KRAB-dCas9 and identify downstream effects on gene expression and cell fate.

The reasons for variable expression from a “safe harbor”-locus are not entirely clear. Silencing has often been observed (and less often published) for transgenes in general^65^ and for transgenes similar to our KRAB-dCas9 construct inserted in the AAVS1 locus.^66^^,^^67^ We find differences in KRAB-dCas9 expression among clones to be fairly stable across passages, although more systematic investigations would be needed to quantify this more precisely. In contrast, differences in KRAB-dCas9 expression among cells within one clone are not stable across passages, as cells sorted for high KRAB-dCas9 expression can revert back to a heterogenous population within one passage (Figure S1D). A negative aspect of this finding is that sorting cells for similar KRAB-dCas9 expression seems not a viable option to generate lines with similar KRAB-dCas9 expression. A positive aspect of this finding is that the switching from a transcribed KRAB-dCas9 transgene to a silenced transgene is fast. Indeed, it seems fast enough to make the magnitude of knockdown efficiency largely independent of KRAB-dCas9 expression levels (Figure 3C). We also observe that the two cynomolgus lines have lower KRAB-dCas9 expression levels than the human and gorilla lines, but whether this is a property of the species would require considerably more clones from more individuals per species. Whether alternative loci and/or insulating DNA elements could decrease variation of KRAB-dCas9 expression levels across species would also be worth exploring in the future.^65^ However, while it is clearly desirable to understand and reduce variable transgene expression at the AAVS1 locus, the decisive criterion for comparable CRISPRi perturbation is the comparable downregulation of the targeted genes across clones and species. At least for our experimental setups and range of KRAB-dCas9 expression levels, the target gene downregulation does not depend strongly on the observed KRAB-dCas9 expression variability. This is good news for CRISPRi approaches in different iPSCs within and across species.

Comparable and efficient downregulation does strongly depend on the targeted gene and on the transduced gRNAs, especially for CRISPRi.^28^^,^^68^ Designing comparably efficient gRNAs across species is another challenge for CRISPR-based comparative approaches, but it is encouraging that we observe an overall comparable and efficient downregulation in our human and cynomolgus KRAB-dCas9 iPSCs. However, the magnitude of downregulation does vary among iPSC lines of the same species and a careful characterization of cell lines and a sufficient number of biological replicates are needed to identify consistent differences between species.^16^^,^^18^ Our results and resources will help to explore such cross-species comparisons by single-cell CRISPR screens to investigate human biology, disease, and evolution.

### Limitations of the study

The fluctuations of KRAB-dCas9 expression between cells from one clone and within cells of a clone are not fully elucidated. We hypothesize that these variations could be influenced by the cell cycle or activation-/deactivation of expression at the locus, however, this remains to be investigated further. While our study suggests that variable expression levels of KRAB-dCas9 are not a major obstacle when comparing CRISPRi screens among iPSC lines, this is probably not true for all genes. Downregulation of some genes might be affected substantially by expression levels of KRAB-dCas9 and/or small expression differences in downregulation might have significant downstream effects. Hence, variable expression levels of KRAB-dCas9 and additionally the well-known variability among clones,^69^^,^^70^ individuals, and species, would still require to screen or at least validate results from CRISPRi screens in a sufficient number of cell lines to generalize findings within a species and across species.

## STAR★Methods

### Key resources table

**Table:** 

REAGENT or RESOURCE	SOURCE	IDENTIFIER
**Antibodies**		

Anti-mouse-IgG Sheep Antibody (HRP (Horseradish Peroxidase))	Cytiva	Cat# NA931; RRID: AB_772210
Anti-Rabbit-IgG Donkey Polyclonal Antibody (HRP (Horseradish Peroxidase))	Cytiva	Cat# NA934; RRID: AB_772206
Donkey anti-Mouse IgG (H + L) Highly Cross-Adsorbed Secondary Antibody, Alexa Fluor™ 488	Thermo Fisher Scientific	Cat# A-21202; RRID: AB_141607
Goat anti-Rabbit IgG (H + L) Highly Cross-Adsorbed Secondary Antibody, Alexa Fluor™ 488	Thermo Fisher Scientific	Cat# A-11034; RRID: AB_2576217
HA-Tag (C29F4) Rabbit mAb #3724	Cell Signaling Technology	Cat# 3724S; RRID: AB_1549585
Oct-4 Rabbit Antibody	Cell Signaling Technology	Cat# 2750S; RRID: AB_823583
Sox2 (L1D6A2) Mouse mAb	Cell Signaling Technology	Cat# 4900S; RRID:AB_10560516
SSEA4 (MC813) Mouse mAb	Cell Signaling Technology	Cat# 4755S; RRID: AB_1264259
β-Actin (8H10D10) Mouse mAb	Cell Signaling Technology	Cat# 3700S; RRID: AB_2242334

**Bacterial and virus strains**		

NEB Stable Competent *E.coli*	New England BioLabs	Cat# C3040I

**Biological samples**		

Cynomolgus Dermal Fibroblast	PELOBiotech	PB-CY-423-0811

**Chemicals, peptides, and recombinant proteins**		

2-Mercaptoethanol	Sigma-Aldrich	Cat# M3148-100ML
Accumax™ cell detachment solution	Sigma-Aldrich	Cat# SCR006
Bovine Serum Albumin (BSA)	Sigma-Aldrich	Cat# A3059-100G
DAPI 4′,6-Diamidine-2′-phenylindole dihydrochloride	Sigma-Aldrich	Cat# 10236276001
DMEM High Glucose	TH. Geyer	Cat# L0102
Doxycycline hyclate	VWR	Cat# J60579.14
DPBS w/o Calcium w/o Magnesium	TH. Geyer	Cat# L0615-500
Ethylenediamine tetraacetic acid (EDTA)	Carl Roth	Cat# CN06.3
Fetal Bovine Serum, qualified, heat inactivated, Brazil (FBS)	Thermo Fisher Scientific	Cat# 10500064
Geltrex™ LDEV-Free, hESC-Qualified, Reduced Growth Factor Basement Membrane Matrix	Thermo Fisher Scientific	Cat# A1413301
Geneticin™ Selective Antibiotic (G418 Sulfate)	Thermo Fisher Scientific	Cat# 11811023
GlutaMAX Supplement	Thermo Fisher Scientific	Cat# 35050038
MEM Non-Essential Amino Acids Solution (100X)	Thermo Fisher Scientific	Cat# 11140035
Paraformaldehyde (PFA)	Sigma-Aldrich	Cat# 441244-1KG
Penicillin-Streptomycin (10.000 U/ml) (PS)	Thermo Fisher Scientific	Cat# 15140122
Proteinase K solution	Thermo Fisher Scientific	Cat# AM2548
Puromycin dihydrochloride	Sigma-Aldrich	Cat# P8833-10MG
QIAzol Lysis Reagent	Qiagen	Cat# 79306
Recombinant Human FGF-basic	PeproTech	Cat# 100-18B
ROTI®Mark TRICOLOR	Carl Roth	Cat# 8271.1
StemFit® Basic02	Nippon Genetics	Cat# 3821.00
StemFit® Basic03	Nippon Genetics	Cat# Basic03
Triton X-100	Sigma-Aldrich	Cat# T8787-50ML
Trypsin-EDTA (0.25%), phenol red	Thermo Fisher Scientific	Cat# 25200072
Y-27632, Dihydrochloride Salt (Rock Inhibitor)	Biozol	Cat# BYT-ORB153635
Buffer RLT Plus	Qiagen	Cat# 1053393

**Critical commercial assays**		

5′ CRISPR Kit, 16 rxns	10x Genomics	Cat# 1000451
Chromium Next GEM Chip K Single Cell Kit, 16 rxns	10x Genomics	Cat# 1000287
Chromium Next GEM Single Cell 5′ Kit v2, 16 rxns	10x Genomics	Cat# 1000263
Direct-zol RNA MicroPrep	Zymo Research	Cat# R2062
DirectPCR® DNA extraction system	VWR	Cat# 732-3255
Dual Index Kit TT Set A, 96 rxns	10x Genomics	Cat# 1000215
DreamTaq Green DNA Polymerase	Thermo Fisher Scientific	Cat# EP0712
ECL™ Western Blotting Detection Reagents	TH. Geyer	Cat# RPN2209
Human Stem Cell Nucleofector™ Kit 2	Lonza	Cat# VPH-5022
Lipofectamine™ 3000 Transfection Reagent	Thermo Fisher Scientific	Cat# L3000015
Maxima Reverse Transcriptase	Thermo Fisher Scientific	Cat# EP0742
NEBuilder HiFi DNA Assembly Master Mix	New England BioLabs	Cat# E2621L
Novex™ WedgeWell™ 8 to 16%, Tris-Glycin, 1.0 mm, Mini Protein Gels	Thermo Fisher Scientific	Cat# XP08165BOX
Pierce™ BCA Protein Assay Kit	Thermo Fisher Scientific	Cat# 23225
PowerUp SYBR Green Master Mix	Thermo Fisher Scientific	Cat# A25742
Bolt™ 4 to 12%, Bis-Tris, 1.0 mm, Mini Protein Gels	Thermo Fisher Scientific	Cat# NW04122BOX
NuPAGE™ Sample Reducing Agent (10X)	Thermo Fisher Scientific	Cat# NP0009
20X Bolt™ MOPS SDS Running Buffer	Thermo Fisher Scientific	Cat# B0001
Restore™ PLUS Western Blot Stripping Buffer	Thermo Fisher Scientific	Cat# 46430
4X Bolt™ LDS Sample Buffer	Thermo Fisher Scientific	Cat# B0007
10X Bolt™ Sample Reducing Agent	Thermo Fisher Scientific	Cat# B0009
NuPAGE™ LDS Sample Buffer (4X)	Thermo Fisher Scientific	Cat# NP0007
Maxima H Minus Reverse Transcriptase	Thermo Fisher Scientific	Cta# EP0753
KAPA HiFi HotStart ReadyMix	Roche	Cat# 07958935001
Exonuclease I	Thermo Fisher Scientific	Cat# EN0581
NEBNext® Ultra™ II FS DNA Library Prep Kit for Illumina	New England BioLabs	Cat# E7805S
BsmBI-v2	New England BioLabs	Cat# R0739S

**Deposited data**		

Raw and processed single-cell RNA-seq data	GEO	GSE241293
Raw and processed bulk RNA-seq data	GEO	GSE255980

**Experimental models: Cell lines**		

wild-type human iPSCs	lab-owned (Geuder et al.^17^); see Table S1	N/A
wild-type gorilla iPSCs	lab-owned (Geuder et al.^17^); see Table S1	N/A
wild-type cynomolgus iPSCs	this paper; see STAR Methods and Table S1	N/A

**Oligonucleotides**		

Oligonucleotides	see Table S2 for oligonucleotides used in this study	N/A
gRNAs	see Table S3 for gRNAs used in this study	N/A

**Recombinant DNA**		

CROP-seq-opti	Addgene	#106280; RRID: Addgene_106280
pAAVS1-NDi-CRISPRi (Gen1)	Addgene	#73497; RRID: Addgene_73497
pAAVS1-TetOn-dCas9-KRAB	gift from R. Maehr^34^ (also available on addgene)	#115545; RRID: Addgene_115545
pCAG-eCas9-GFP-U6-gRNA RhAAVS1-v2	gift from C. E. Dunbar^62^	N/A
pHAGE-EF1a-AAVSZnFG-PGK_puro	gift from R. Maehr	N/A
pLX303-ZIM3-KRAB-dCas9	Addgene	#154472; RRID: Addgene_154472
pMD2.G	Addgene	#12259; RRID: Addgene_12259
pMDLg/pRRE	Addgene	#12251; RRID: Addgene_12251
pRSV-Rev	Addgene	#12253; RRID: Addgene_12253
pAAVS1-TetOn-ZIM3-KRAB-dCas9-P2A-mCherry	this paper, Addgene	Addgene #212829
pCyno-AAVS1-TetOn-ZIM3-KRAB-dCas9-P2A-mCherry	this paper, Addgene	Addgene #212830
pGorilla-AAVS1-TetOn-ZIM3-KRAB-dCas9-P2A-mCherry	this paper, Addgene	Addgene #212831

**Software and algorithms**		

afex	https://cran.r-project.org/package=afex	Version 1.3–0
Biostrings	https://bioconductor.org/packages/Biostrings/	Version 2.66.0
Cellranger	10x Genomics	Version 7.0.0
cellsnp-lite	https://cellsnp-lite.readthedocs.io/en/latest/	Version 1.2.2
cowplot	https://cran.r-project.org/package=cowplot	Version 1.1.1
doParallel	https://cran.r-project.org/package=doParallel	Version 1.0.17
dplyr	https://dplyr.tidyverse.org/	Version 1.1.2
FlowJo V10.8.2	FlowJo	663441
foreach	https://cran.r-project.org/package=foreach	Version 1.5.2
gelnet	https://cran.r-project.org/package=gelnet	Version 1.2.1
ggbeeswarm	https://cran.r-project.org/package=ggbeeswarm	Version 0.7.2
ggnewscale	https://cran.r-project.org/package=ggnewscale	Version 0.4.9
ggplot2	https://ggplot2.tidyverse.org/	Version 3.4.2
ggplotify	https://cran.r-project.org/package=ggplotify	Version 0.1.1
ggrepel	https://cran.r-project.org/package=ggrepel	Version 0.9.3
ImageJ (Fiji)	https://imagej.net/ij/	Version 2.0.0-rc-69/1.52n
lattice	https://cran.r-project.org/package=lattice	Version 0.21–8
limma	https://bioconductor.org/packages/limma/	Version 3.56.2
lmerTest	https://cran.r-project.org/package=lmerTest	Version 3.1–3
Matrix	https://cran.r-project.org/package=Matrix	Version 1.6–0
multcomp	https://cran.r-project.org/package=multcomp	Version 1.4–25
patchwork	https://patchwork.data-imaginist.com/	Version 1.1.2
plyranges	https://bioconductor.org/packages/plyranges/	Version 1.18.0
R	R Foundation for Statistical Computing, Vienna, Austria	Version 4.1.3
RColorBrewer	https://cran.r-project.org/package=RColorBrewer	Version 1.1–3
readr	https://readr.tidyverse.org/	Version 2.1.4
scater	https://bioconductor.org/packages/scater/	Version 1.28.0
scran	https://bioconductor.org/packages/scran/	Version 1.28.2
scuttle	https://bioconductor.org/packages/scuttle/	Version 1.8.4
Seurat	https://satijalab.org/seurat/	Version 4.9.9.9058
SeuratObject	https://cran.r-project.org/package=SeuratObject	Version 4.9.9.9091
SingleCellExperiment	https://bioconductor.org/packages/SingleCellExperiment/	Version 1.20.1
SingleR	https://bioconductor.org/packages/SingleR/	Version 2.0.0
stringr	https://stringr.tidyverse.org/	Version 1.5.0
tibble	https://tibble.tidyverse.org/	Version 3.2.1
tidyr	https://tidyr.tidyverse.org/	Version 1.3.0
tidyseurat	https://cran.r-project.org/package=tidyseurat	Version 0.6.1
transformGamPoi	https://bioconductor.org/packages/transformGamPoi/	Version 1.4.0
vireoSNP	https://vireosnp.readthedocs.io/en/latest/	Version 0.5.7
zUMIs	https://github.com/sdparekh/zUMIs	Version 2.9.4days
fastQC	https://www.bioinformatics.babraham.ac.uk/projects/fastqc/	
Cutadapt	https://cutadapt.readthedocs.io/en/stable/	
DESeq2	https://bioconductor.org/packages/release/bioc/html/DESeq2.html	

**Other**		

10x Chromium Controller	10x Genomics	
Bioanalyzer High Sensitivity DNA Analysis	Agilent	5067–4626
Countess™ II automated cell counter	Thermo Fisher Scientific	AMQAX1000
Immobilon-PSQ PVDF Membrane	Merck Millipore	ISEQ15150
Microscope Nikon eclipse TE2000-S	Nikon	TE20000-S
Nucelofector 2b Device	Lonza	AAB-1001

### Resource availability

#### Lead contact

Further information and requests for resources and reagents should be directed to and will be fulfilled by the lead contact, Wolfgang Enard (enard@bio.lmu.de).

#### Materials availability

Plasmids generated in this study have been deposited to (Addgene, pAAVS1-TetOn-ZIM3-KRAB-dCas9-P2A-mCherry, #212829; Addgene, pCyno-AAVS1-TetOn-ZIM3-KRAB-dCas9-P2A-mCherry, #212830; Addgene, pGorilla-AAVS1-TetOn-ZIM3-KRAB-dCas9-P2A-mCherry, #212831).

#### Data and code availability

• Single-cell RNA-seq data and bulk RNA-seq data have been deposited at GEO and are publicly available as of the date of publication. Accession numbers are listed in the key resources table. Other data reported in this paper will be shared by the lead contact upon request.
• This paper does not report original code.
• Any additional information required to reanalyze the data reported in this paper is available from the lead contact upon request.

### Experimental model and study participant details

#### Cell lines

We report growth conditions of the cell lines in the Method details and listed information about the cell lines in Table S1.

### Method details

#### Assembly of the AAVS1-targeting KRAB-dCas9-coding donor plasmids

Using Gibson assembly, we modified the human AAVS1 locus targeting pAAVS1-TetOn-dCas9-KRAB^34^ (RRID:Addgene_115545, gift from M. Ziller and R. Maehr). We replaced the dCas9-KRAB CDS with a ZIM3-KRAB-dCas9-HA-NLS sequence, amplified from pLX303-ZIM3-KRAB-dCas9^26^ (gift from Mikko Taipale; RRID:Addgene_154472), to create our first version of the donor plasmid, the pAAVS1-TetOn-ZIM3-KRAB-dCas9. To add mCherry to this construct, we combined our first version with the pAAVS1-NDi-CRISPRi (Gen1)^46^ (gift from Bruce Conklin; RRID:Addgene_73497), by DraIII-digest and ligation. Lastly, we introduced a Gly-Ser-Gly-CDS before the P2A to produce the final human donor plasmid, pAAVS1-TetOn-ZIM3-KRAB-dCas9-P2A-mCherry (Addgene, #212829).

For cloning of the non-human primate AAVS1 targeting plasmids, we obtained the AAVS1 orthologous regions of gorilla and cynomolgus by UCSC BLAT search and amplified the regions from genomic DNA that we extracted from primate wt iPSCs. Every non-human primate AAVS1 right and left homology arms (HA-R and HA-L) were assembled with a ampR- and oriP-containing minimal vector with a bridging fragment bearing a AscI-restriction-site, to link and circularize HA-L and HA-R, resulting in pGorilla-LOR and pCyno-LOR.

To assemble the final cynomolgus donor plasmid, the human donor plasmid (pAAVS1-TetOn-ZIM3-KRAB-dCas9-P2A-mCherry) was combined with the AscI-digested pCyno-LOR to create the final pCyno-AAVS1-TetOn-ZIM3-KRAB-dCas9-P2A-mCherry (Addgene, #212830). Here, AscI restriction sites flanking the TetOn-ZIM3-KRAB-dCas9-P2A-mCherry cassette were preserved. For the gorilla donor plasmid, the final cynomolgus donor plasmid pCyno-AAVS1-TetOn-ZIM3-KRAB-dCas9-P2A-mCherry and the pGorilla-LOR were combined by AscI-digest and subsequent ligation, to create the final gorilla donor plasmid, pGorilla-AAVS1-TetOn-ZIM3-KRAB-dCas9-P2A-mCherry (Addgene, #212831) (see also Figure S6).

#### Generation of cynomolgus iPSCs with SeV transduction

Cynomolgus dermal fibroblasts (PELOBiotech, PB-CY-423-0811) were reprogrammed using the CytoTune-iPS 2.0 Sendai Reprogramming kit (Thermo Fisher Scientific, A16518) as previously described.^17^^,^^41^^,^^59^ Briefly, 70,000 cells were resuspended in 100 μL of SeV mix containing polycistronic Klf4-Oct3/4-Sox2, cMyc and Klf4 at a MOI of 5. Afterward, the cells were incubated with the virus for 1 h at 37°C for suspension infection, before seeding in a 1% Geltrex-coated (Thermo Fisher Scientific, A1413301) 12-well. The SeV-containing medium was exchanged by fresh fibroblast culture medium (DMEM High Glucose (TH Geyer, L0102) supplemented with 10% FBS (Thermo Fisher Scientific, 10500064) and 1% Pen/Strep (Thermo Fisher Scientific, 15140122)) the next day. On day 5 after transduction, the medium was changed to mTesR1 (STEMCELL Technologies, 05850) with medium changes every other day. Emerging iPSC colonies were manually picked and seeded on 1% Geltrex-coated plates in StemFit Basic02 (Nippon Genetics, Basic02) supplemented with 100 ng/mL bFGF (Preprotech, 100-18B) and 1% Pen/Strep.

#### Cell maintenance

Human and gorilla iPSCs were generated and validated by our group as described in Geuder et al.^17^ and Radmer et al.^41^; cynomolgus iPSCs were generated as described above. All iPSCs were maintained in StemFit03 (Nippon Genetics, Basic03) supplemented with 100 ng/mL bFGF and 1% Pen/Strep on 1% Geltrex-coated wells of a 12-well plate. Medium was changed every other day and iPSCs were split in clumps every 4–5 days using 0.5 mM EDTA (Carl Roth, CN06.3). KRAB-dCas9 iPSCs were cultured in the same manner.

HEK293T cells were maintained in DMEM High Glucose (TH Geyer, L0102), supplemented with 10% FBS, 1% GlutaMAX (Thermo Fisher Scientific, 35050038), 1% non-essential amino acids (Thermo Fisher Scientific, 11140035), and 1% Pen/Strep. HEK293T cells were split every 2–3 days with 0.25% Trypsin-EDTA (Thermo Fisher Scientific, 25200072).

#### Generation of KRAB-dCas9 iPSCs

Wt human, gorilla and cynomolgus iPSCs were dissociated to single cells using Accumax (Sigma-Aldrich, SCR006) and 1x10^6^ cells were pelleted at 300 g for 5 min. Nucleofection reagents were prepared according to the manufacturer’s instructions (Lonza, VPH-5022). 3 μg of the donor plasmid encoding the KRAB-dCas9-cassette and 7 μg of a nuclease-encoding plasmid (ZnF-encoding plasmid targeting the human and gorilla AAVS1 locus, pHAGE-EF1a-AAVSZnFG-PGK_puro, was a gift from Rene Maehr;^34^ eCas9-encoding plasmid targeting the cynomolgus AAVS1 locus, pCAG-eCas9-GFP-U6-gRNA RhAAVS1-v2, was a gift from Cynthia E. Dunbar^73^), were added to the nucleofection-mix. Nucleofection was performed using the B016 setting of the Nucleofector 2b Device (Lonza, VPH-022). Next, 1x10^6^ nucleofected iPSCs were seeded into two 1% Geltrex-coated wells of a 6-well plate in StemFit03 supplemented with 10 μM Y-27632 (Biozol, BYT-ORB153635). 48 h after nucleofection, cells were selected with 100–150 μg/mL G418 (Thermo Fisher Scientific, 11811023) for 7–10 days. After selection, colonies were picked and expanded in 1% Geltrex-coated wells of a 24-well plate. Total gDNA was isolated from cell pellets using the DirectPCR DNA extraction system (VWR, 732–3255) with supplemented Proteinase K solution (Thermo Fisher Scientific, AM2548) at 55°C and 550 rpm for 5 min and 85°C for 45 min.

For genotyping of the knock-in AAVS1 locus, PCRs for the gorilla clones and nested PCRs for the human and cynomolgus clones were performed (Gorilla PCR: p205, p271; Human PCR1: p205, p188, PCR2: p186, p277; Cynomolgus PCR1: p205, p276, PCR2: p186, p272). Furthermore, primers binding to the AAVS1 locus were used for identification of a wt locus (Human: p83, p84; Gorilla: p84, p273, Cynomolgus: p83, p84). The sequences of the used primers can be found in Table S2. All PCRs were performed using the Green DreamTaq Polymerase (Thermo Fisher Scientific, EP0712).

#### Immunofluorescence stainings

KRAB-dCas9 iPSCs were cultured in SF03 medium without or with 1 μg/mL dox (VWR, J60579.14) for 4 days. Medium was changed every other day. Cells were fixed with 4% PFA (Sigma-Alrich, 441244-1KG) for 15 min at room temperature (RT). Fixed cells were blocked and permeabilized with DPBS (TH. Geyer, L0615-500) containing 5% FBS and 0.3% Triton X-100 (Sigma-Aldrich, T8787-50ML) for 30 min at RT. All antibodies were diluted in DPBS with 1% BSA (Sigma-Aldrich, A3059-100G) and 0.3% Triton X-100. The cells were incubated with the primary antibodies SSEA4 (1:500; Cell Signaling Technology 4755, RRID:AB_1264259), OCT-4 (1:400; Cell Signaling Technology 2750, RRID:AB_823583), and HA-tag (1:1000; Cell Signaling Technology 3724, RRID:AB_1549585) overnight at 4°C. Cells were washed three times with DPBS and then incubated with secondary anti-mouse Alexa Fluor 488 (1:500; Thermo Fisher Scientific A-21202, RRID:AB_141607) or anti-rabbit Alexa Fluor 488 (1:500; Thermo Fisher Scientific A-11034, RRID:AB_2576217) antibodies for 1 h at RT in the dark. Counterstaining was performed using 1 μg/mL DAPI (Sigma-Aldrich, 10236276001) for 1 min and cells were washed with DPBS three times for 5 min. Images were obtained using a Nikon eclipse TE2000-S microscope (Nikon, TE2000-S) and edited using Fiji (Version 2.0.0-rc-69/1.52n).

#### Fluorescence-activated cell sorting

To enrich for pure mCherry-positive populations, human H.i1_clone1, and cynomolgus C.i1_clone1 and C.i2_clone1 were sorted by flow cytometry using a BD FACS Aria III Cell Sorter. Before sorting, KRAB-dCas9 iPSCs were cultured in SF03 with 1 μg/mL dox for three days, dissociated to single cells and transferred to DPBS with 0.5% BSA, 2 mM EDTA and 25 mM HEPES (Sigma-Aldrich, 83264-100ML-F). mCherry-positive cells were sorted into SF03 with 10 μM Y-27632 and 25 mM HEPES. Sorted single cells were seeded in SF03 with 10 μM Y-27632 and then maintained as described above. FACS data was analyzed using the FlowJo Software (V10.8.2).

#### Flow cytometry

After sorting, KRAB-dCas9 iPSCs were cultured for some passages and then analyzed by flow cytometry to compare the mCherry-positive fractions of the clones over time. To do so, KRAB-dCas9 iPSCs were cultured in SF03 medium without or with 1 μg/mL dox for 4 days. Medium was changed every other day. Cells were washed with DPBS and dissociated with Accumax to single cells. Cells were pelleted at 300 g for 5 min at RT, then the cell pellet was resuspended in DPBS with 0.5% Bsa, 0.01% NaN_3_, and 1 μg/mL DNaseI and cells were transferred to round-bottom polystyrene tubes (VWR, 734-0001). Cells were captured on a BD LSRFortessa Cell Analyzer and data was analyzed using the FlowJo Software (V10.8.2).

#### Western Blots

KRAB-dCas9 iPSCs were cultured in SF03 without or with 1 μg/mL dox for 4 days. Medium was changed every other day. Cells were washed with DPBS, collected with a cell scraper (VWR, 734-0385) and pelleted at 300 g for 5 min at RT. The cell pellet was lysed in RIPA buffer (50 mM Tris HCl (pH 7.4), 1% NP-40, 0.25% Sodium deoxycholate, 150 mM EDTA, 1 mM Na_3_VO_4_, 200 μM PMSF, freshly added protease inhibitor cocktail in a 1/10 ratio (v/v)) and incubated on ice for 30 min. Samples were vortexed and centrifuged at 20,000 g for 30 min at 4°C. The supernatant was collected in a new tube and protein concentrations were quantified with a BCA protein assay kit (Thermo Fisher Scientific, 23225). 15 μg protein per sample was run on precast Novex WedgeWell 8 to 16% Tris-Glycine gels (Thermo Fisher Scientific, XP08165BOX), using NuPAGE Sample Reducing Agent (10X, Thermo Fisher Scientific, NP0009) and NuPAGE LDS Sample Buffer (4X, Thermo Fisher Scientific, NP0007). Proteins were transferred to a PVDF membrane (Merck Millipore, ISEQ15150) overnight at 4°C, then membranes were blocked with 5% skimmed milk in TBS-T. For KRAB-dCas9 detection proteins were stained with an anti-HA (1:1,000; Cell Signaling Technology 3724, RRID:AB_1549585), or anti-beta-Actin (1:1,000; Cell Signaling Technology 3700, RRID:AB_2242334) primary antibody for 2 h at RT. Membranes were washed three times with TBS-T and incubated with an HRP-coupled anti-rabbit (1:10,000; Cytiva NA934, RRID:AB_772206), or HRP-coupled anti-mouse (1:10,000; Cytiva NA931, RRID:AB_772210) secondary antibody for 1 h at RT. After antibody incubation, membranes were washed three times with TBS-T and proteins were detected with ECL western blotting detection reagents (TH. Geyer, RPN2209) and imaged with a BioRad ChemiDoc MP Imaging System. The protein signal was quantified with Fiji (Version 2.0.0-rc-69/1.52n) and the KRAB-dCas9 signal was normalized to the beta-Actin signal.

#### *SOX2*-perturbation assay

A *SOX2*-targeting gRNA published by Mandegar et al.^46^ was cloned into the CROP-seq-opti^47^ vector (gift from Jay Shendure; RRID:Addgene_106280) using a published protocol by Datlinger et al.^31^ Briefly, gRNAs were cloned into the BsmBI-digested (New England BioLabs, R0739S) CROP-seq-opti vector by Gibson Assembly using the NEBuilder HiFi DNA Assembly Master Mix (New England BioLabs, E2621L) and NEB Stable Competent *E. coli* (New England BioLabs, C3040I) were transformed with the assembled plasmids. Lentivirus particles were produced following the same published protocol.^31^ In short, HEK293T cells were transfected with the SOX2-gRNA-CROP-seq-opti plasmid and three packaging plasmids: pMDLg/pRRE (RRID:Addgene_12251), pRSV-Rev (RRID:Addgene_12253) and pMD2.G (RRID:Addgene_12259) using the lipofectamine 3000 transfection reagent (Thermo Fisher Scientific, L3000015). Medium was exchanged 6 h after transfection and lentivirus was harvested 24 h and 48 h after transfection. For transduction, KRAB-dCas9 iPSCs were dissociated to single cells using Accumax and incubated in lentivirus containing medium supplemented with 10 μM Y-27632 for 1 h at 37°C, before seeding into 1% Geltrex-coated wells. Non-transduced cells were treated in the same manner, except no virus was added to the medium. Medium was exchanged for fresh medium supplemented with 10 μM Y-27632 after 24 h. 2 days after transduction, transduced cells were selected with 1.3 μg/mL puromycin (Sigma-Aldrich, P8833-10MG) for 3 days. Transduced KRAB-dCas9 iPSCs with a stable integrated SOX2-gRNA were cryopreserved for further experiments.In a separate experiment, transduced and non-transduced cell stocks were thawed and cultured in SF03 without or with 1 μg/mL dox for 4 days. After that, cells were lysed in QIAzol Lysis Reagent (Qiagen, 79306). Total RNA was isolated using the Direct-zol RNA MicroPrep kit (Zymo Research, R2062) according to the manufacturer’s instructions. 250 ng RNA per sample was reverse transcribed using the Maxima Reverse Transcriptase (Thermo Fisher Scientific, EP0742). 5 ng of the cDNA was used for quantitative PCR analysis using target-specific primers (see Table S1) and the PowerUp SYBR Green Master Mix (Thermo Fisher Scientific, A25742). Transcripts were normalized to GAPDH expression and relative *SOX2* transcript expression was determined between +dox and -dox samples using the ΔΔCt method.

Immunofluorescence stainings of KRAB-dCas9 iPSCs with a *SOX2*-targeting gRNA, cultured in +dox- or -dox-containing medium, were performed as described above using a SOX primary antibody (1:400; Cell Signaling Technologies, 4900S; RRID:AB_10560516) and anti-mouse Alexa Fluor 488 secondary antibody.

Western Blots for SOX2 were performed as described above, but using Bolt 4 to 12%, Bis-Tris gels (Thermo Fisher Scientific, NW04120BOX) in combination with 20X Bolt MOPS SDS Running Buffer (Thermo Fisher Scientific, B0001), 4X Bolt LDS Sample Buffer (Thermo Fisher Scientific, B0007) and 10X Bolt Sample Reducing Agent (Thermo Fisher Scientific, B0009). Furthermore, first SOX2 was detected with an anti-SOX2 primary antibody (1:1,000; Cell Signaling Technologies, 4900S; RRID:AB_10560516) and an HRP-coupled anti-mouse secondary antibody. Then antibodies were removed using Restore PLUS Western Blot Stripping Buffer (Thermo Fisher Scientific, 46430) and protein detection for beta-Actin was performed as described above.

#### Bulk RNA-sequencing

KRAB-dCas9 iPSCs that were previously transduced with a *SOX2*-targeting gRNA (as described above) were cultured in 24-wells in SF03 medium with or without 1 μg/mL dox for 4 days. Medium was changed every other day. Cells were washed with PBS and lysed in 200 μL Buffer RLT Plus (Qiagen, 1053393) supplemented with 1% 2-Mercaptoethanol (Sigma-Aldrich, M3148-100ML) and stored at −80°C until processing. cDNA synthesis and library generation were performed as described in the prime-seq method.^48^ The detailed protocol, along with primer sequences, is available on protocols.io (https://www.protocols.io/view/prime-seqs9veh66). In brief, cDNA synthesis was conducted using Maxima H Minus Reverse Transcriptase (Thermo Fisher Scientific, EP0753), oligo-dT primer E3V7NEXT, and a custom template-switching oligo. After pooling the cDNA of all processed samples, primers were removed using Exonuclease I (Thermo Fisher Scientific, EN0581) and cDNA pre-amplification was performed using KAPA HiFi HotStart polymerase (Roche, 07958935001) and the custom SingV6 primer. Next, libraries were constructed from 8.7 ng of total cDNA with the NEBNext Ultra II DNA Library Prep Kit for Illumina (New England Biolabs, E7805S) with a custom ligation-adapter and TruSeq i5 and Nextera i7 primers for dual-indexing. Libraries were sequenced on an Illumina NextSeq 1000/2000 with the following parameters: read 1 (28 bases), read 2 (8 bases), read 3 (8 bases), and read 4 (93 bases). Fastq data file quality assessment was conducted using fastqc,^74^ and polyA trimming was performed with cutadapt.^75^ Quality filtering, mapping, and counting was done with the zUMIs pipeline.^76^ For mapping we used the reference genomes *Homo sapiens* hg38 (GENCODE release 32), *Gorilla gorilla* Kamilah_GGO_v0/gorGor6 (UCSC, Aug. 2019), and *Macaca fascicularis* macFas6 (ENSEMBL, Mar. 2020). Furthermore, all reference genomes were extended by the sequence of the respective KRAB-dCas9-coding donor plasmid for each species. The gorilla and macaque gene annotation GTF file was created by liftoff^77^ of the hg38 annotation to the gorGor6 or macFas6 genome. Normalization, variance stabilization, and principal component analysis of the top 500 variable genes was done with DESeq2^78^ employing the design model: Y_(g) ∼ Dox + Clone + Dox:Clone. To evaluate the log2FC of *SOX2* expression between conditions with and without dox for each clone separately, DESeq2 was rerun using a concatenated design of clone and dox condition. Lastly, we used the normalized counts to perform cell type classification with SingleR^49^ using a published dataset from Rhodes et al.^50^ as a reference. We filtered the correlation scores of our samples to the reference data for a min. score of 0.5 for a cell type in either the +dox or the minus dox condition. Based on this, all correlation scores to Neurons and Epithelial_Cells were not further included. Then we determined the Δscore for the cell types between the +dox score and -dox score of each clone. Furthermore, we used the SingleR-generated cell label, which assigns each sample with a cell type, indicating the highest correlation between the sample and the reference.

#### Single-cell CRISPR screen

We selected 24 transcription factors and designed gRNAs to target these genes in human and cynomolgus iPSCs using an adaptation of the machine learning-based CRISPRi/a design tool by Horlbeck et al.^68^ We designed species-specific gRNA-libraries by selecting four gRNAs for each targeted promoter that had comparably high predicted activities across the two species (Table S3, ordered as two separate IDT oPools). For each species, we cloned the gene targeting gRNAs and 15 non-targeting gRNAs in a pooled format in the CROP-seq-opti vector^47^ and produced lentivirus particles as described above and in Datlinger et al.^31^ Human (H.i1_clone1 and H.i2_clone2) and cynomolgus (C.i1_clone1) KRAB-dCas9 iPSCs were transduced with their respective gRNA lentiviral library with a MOI of 0.1 and then selected with 1.3 μg/mL puromycin for 3 days. After selection, the transduced cells were cultured in medium with 1 μg/mL dox for 5 days. Medium was changed every other day and the cells were split once with 0.5 mM EDTA after 2 days of dox-induction. After 5 days, single cells were harvested to generate 5′ single-cell gene-expression (GEX) libraries and CRISPR libraries capturing the gRNAs using the 10x Genomics platform (10x Genomics, PN-1000287, PN-1000451, PN-1000263, PN-1000215) with a targeted cell recovery of 16,000 cells. GEX libraries and CRISPR libraries were pooled 4:1 and sequenced on a NextSeq1000/1500 with a 100-cycle kit and the following sequencing setup: read 1 (28 bases), read 2 (8 bases), read 3 (8 bases), and read 4 (93 bases).

Reads were processed using 10x CellRanger (Version 7.0.0, https://support.10xgenomics.com/single-cell-gene-expression/software/pipelines/latest/what-is-cell-ranger). We mapped the CRISPR library to a custom reference, created using the gRNA protospacer sequences and the GEX library to the hg38 and macFas6 reference genome as described above. The reads were demultiplexed into 2 donors using cellsnp-lite^79^ and vireo,^80^ then the donors were assigned to species based on the number of mapped reads in each genome. Next, the human reads were demultiplexed into the 2 individuals based on an SNP list compiled from bulk RNA-seq data of the WT cell lines. For further analysis, we only kept cells from the cynomolgus and one human (H.i2_clone2) which passed the QC requirements (>2000 reads, >1100 genes and <7% mitochondrial reads), which had only one dominant gRNA (i.e., all other detected gRNAs in that cell made up <10% of all gRNA UMIs and <1000 UMIs together), and where the dominant gRNA was detected with >10 UMIs. Furthermore, the species-specific library where the gRNA came from needed to match the species assignment of the cell. Next, for each species we removed control gRNAs (non-targeting) that were detected in less than 10 cells. For the remaining control gRNAs, we performed DE testing by limma-trend,^72^ comparing each non-targeting gRNA against all others, and found that in all cases we detected similar low numbers of DE genes. In total, 1297 human and 2115 cynomolgus cells passed all filtering criteria.

To check the downregulation of the target genes, we performed DE analysis between each targeting gRNA and all non-targeting control gRNAs per species. We only analyzed target genes that had a log-normalized expression of at least 0.2 in both human and cynomolgus control cells and gRNAs that had at least 10 perturbed cells. This way, we could test 11 genes and 15 gRNAs in the human and 16 genes and 22 gRNAs in the cynomolgus data. In all cases, we first subsetted the count matrix for a given gRNA and the controls, removed genes that were lowly expressed (<10% of the cells or <5 cells in both conditions), and normalized the counts using scran.^81^ Then we fitted the model ∼ condition (perturbed or control) and calculated contrasts between the perturbed and control cells with empirical Bayes moderation of the standard errors via limma-trend.^72^ We regarded a target gene as significantly downregulated if it had an adjusted *p*-value < 0.1 (one-tailed *p*-values and Benjamini-Hochberg FDR correction across the targets only). For each target gene, we considered the gRNA leading to the greatest absolute log2 fold change of the target as the ‘best gRNA’. We also quantified target downregulation as knockdown percentage by calculating (1 - mean_expr_prtrb/mean_expr_cntrl)∗100 for each gRNA, where mean_expr_prtrb and mean_expr_cntrl are the mean log-expression of the target gene in the perturbed and control cells, respectively.

To assess the downstream effects of a perturbation, we performed DE analysis between perturbed and control cells within and across species for the entire transcriptome. For this, we kept only the control cells and the cells with the best gRNAs for the 7 perturbations (*ADNP, NFATC3, POU5F1, RCOR3, SALL2*, and *TCF4*) where the cell number and expression was high enough to assess target downregulation and we observed a significant effect in both species. In addition, we removed non-protein coding and mitochondrial genes as well as genes that were lowly expressed (<10% of cells or <5 cells in each perturbation and the control cells). Next, we normalized the human and cynomolgus counts together using scran^81^ and *multiBatchNorm* from batchelor.^82^ Then we fitted the model ∼condition + species + condition:species per perturbation using limma-trend^72^ and calculated contrasts between the perturbed and control cells within human (contrast = human), between the perturbed and control cells within cynomolgus (contrast = cynomolgus), and between the perturbed-control differences across the two species (contrast = interaction) with empirical Bayes moderation of the standard errors. We regarded a downstream gene significantly downregulated within a species or significantly differentially regulated across the species if the corresponding contrast had an adjusted *p*-value < 0.05 (two-tailed *p*-values and Benjamini-Hochberg FDR correction). In total, we tested 11301 genes for all perturbations.

For characterization of the pluripotency state of the cells, we calculated a transcriptome-based stemness index for each cell. This is based on a one-class logistic regression model trained on stem cell classes and their differentiated ecto-, meso-, and endoderm progenitors in the Progenitor Cell Biology Consortium (PCBC) Roadmap data.^71^ The scores were scaled to be between 0 and 1 for the two species together.

Finally, we investigated whether the KRAB-dCas9 levels in the cells have an effect on target downregulation. In each species, we studied the same set of targets as in the DE analysis. Using the R-package lmerTest,^83^ we fitted the following linear mixed-effects model per species: target_expr ∼ dCas9_expr + (1|target/gRNA). Here, target_expr is the transformed expression of the target genes in the perturbed and control cells, calculated by the function transformGamPoi^84^ with the transformation ‘randomized_quantile_residuals’ (predicted variable), dCas9_expr is one of the following 6 categories: cntrl (control cells regardless of KRAB-dCas9 expression), 0/1/2/3/4+ (perturbed cells with 0/1/2/3/≥4 KRAB-dCas9 UMI counts (fixed effect)), target is one of the perturbed genes and gRNA is one of the gRNAs designed for this target gene (nested random effect). We compared the perturbed cells with different KRAB-dCas9 level categories against the control cells, as well as the perturbed cells with KRAB-dCas9 levels 1/2/3/4+ against the perturbed cells with KRAB-dCas9 level 0 using two-sided multiple comparisons of means by the function glht from multcomp^85^ (Tukey’s HSD test). We considered contrast coefficients with an adjusted *p*-value < 0.1 as significant.

### Quantification and statistical analysis

All information about statistical details and parameters of the experiments can be found in the respective figure legends, results and method details. Also, the definition of significance levels can be found in the figure legends and methods.

## Supplemental information

Document S1. Figures S1‒S6

Table S1. Information about the iPS cell lines, related to STAR Methods

Table S2. Sequences of the oligos used in this study, related to STAR Methods

Table S3. Protospacer sequences of the gRNAs used in the single-cell CRISPR screen targeting the human (hg38) and cynomolgus (macFas6) genome, related to STAR Methods

Table S4. Downstream targets per perturbed TF, related to Figures 5G and S5D
