# Supplementary material for: Generation and characterization of inducible KRAB-dCas9 iPSCs from primates for cross-species CRISPRi
Source: iScience. 2024 May 23;27(6):110090. doi: 10.1016/j.isci.2024.110090 (PMC11214527; doi:10.1016/j.isci.2024.110090)
Supplement: Document S1. Figures S1‒S6 [file mmc1.pdf]

iScience, Volume *27*

Supplemental information

Generation and characterization of inducible KRAB-dCas9 iPSCs from primates for cross-species CRISPRi

Fiona C. Edenhofer, Anita Térmeg, Mari Ohnuki, Jessica Jocher, Zane Kliesmete, Eva Briem, Ines Hellmann, and Wolfgang Enard

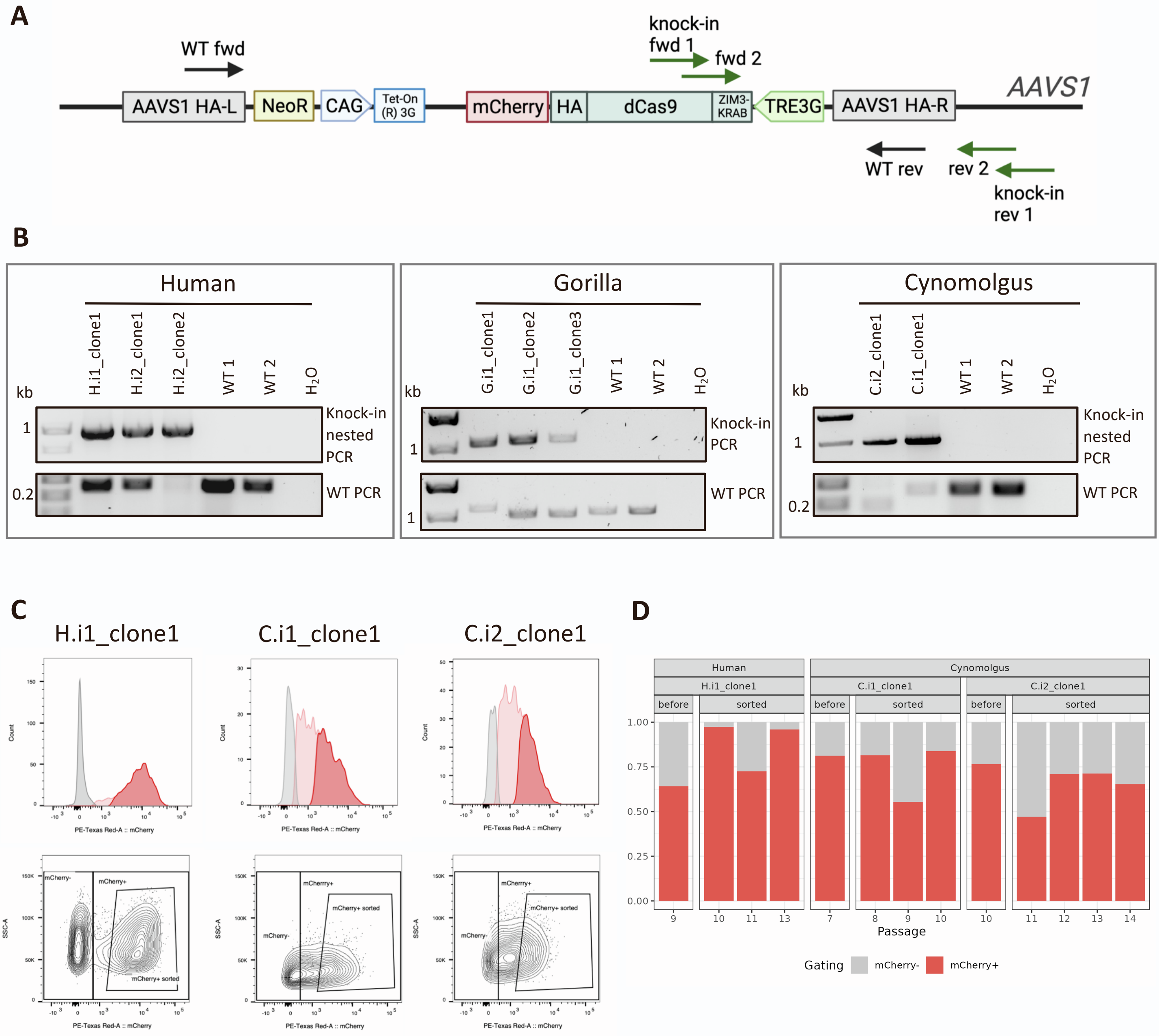

Figure S1. Genotyping and FACS. *Related to Figure 1 and Figure 2.* (**A**) For genotyping after integration, the binding sites for knock-in genotyping primers are indicated as green arrows (knock-in fwd1, fwd2, knock-in rev1, rev2). Wt genotyping primers are shown as black arrows (WT fwd and WT rev). Primer sequences and combinations can be found in Table S2 (Arrows are only shown schematically and do not represent the exact primer binding sites). (**B**) Genotyping of the human and cynomolgus knock-in AAVS1 locus was performed as a nested PCR with species specific knock-in primers fwd/rev1 and fwd/rev2, for the gorilla one PCR was performed. To analyze for a possible wt locus in the clones, species-specific WT fwd and WT rev primers were used for a PCR. This revealed heterozygosity of two human clones, three gorilla clones and two cynomolgus clones. human H.i2_clone2 showed homozygous integration of the construct into the AAVS1 locus. (**C**) H.i1_clone1, C.i1_clone1 and C.i2_clone1 were sorted to enrich for mCherry-positive fractions. Colors and gating show the mCherry-negative, mCherry-positive and the sorted populations. (**D**) mCherry-positive and mCherry-negative fractions in the clones were analyzed by flow cytometry before and ≥3 passages after FACS.

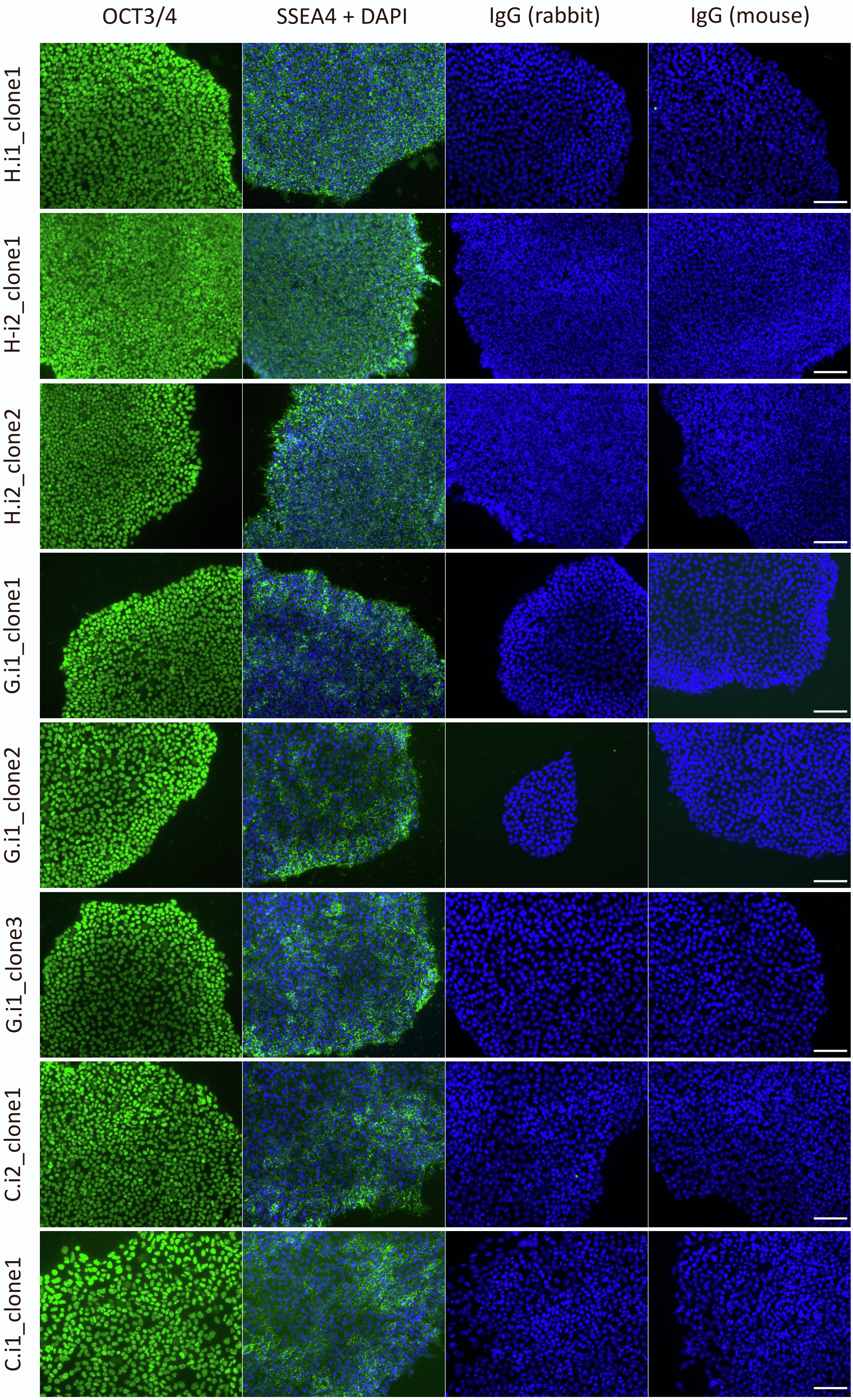

Figure S2. Immunofluorescence stainings to ensure pluripotent state of the knock-in iPSCs. *Related to Figure 1.* Immunofluorescence staining of all presented clones of the pluripotency markers OCT3/4 and SSEA4. Isotype controls are shown for the used antibodies; scale bar represents 100 µm.

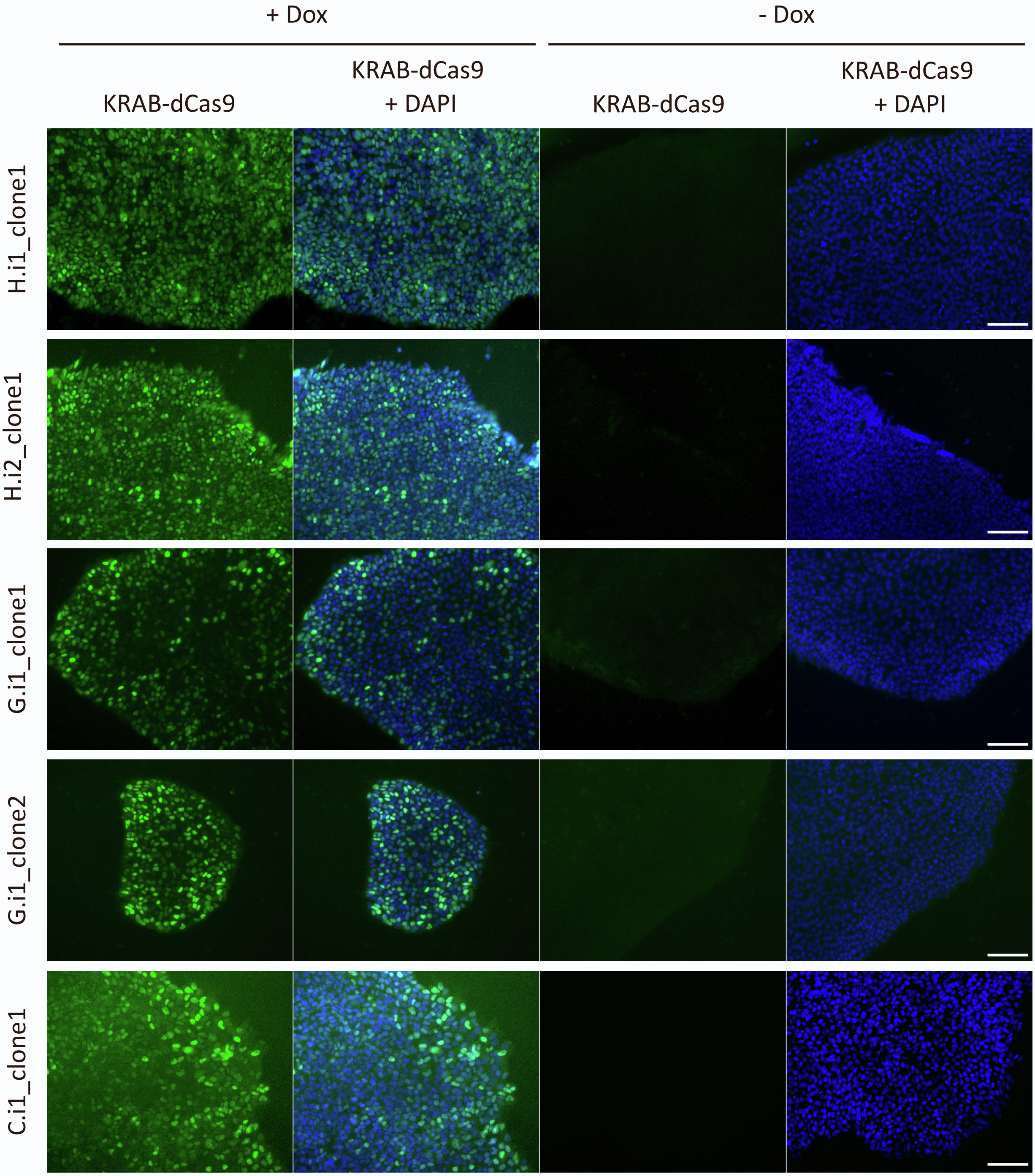

Figure S3. Dox-inducible expression of KRAB-dCas9. *Related to Figure 2.* Immunofluorescence stainings for KRAB-dCas9 (via detection of the fused HA-tag) of humans H.i1_clone1 and H.i2_clone1, gorillas G.i1_clone2 and G.i1_clone2, and cynomolgus C.i1_clone1. Before fixation, KRAB-dCas9 iPSCs were cultured in medium with or without 1 µg/mL dox for 4 days. Isotype controls were performed in the same experiment which is shown in Figure S2; scale bar represents 100 µm.

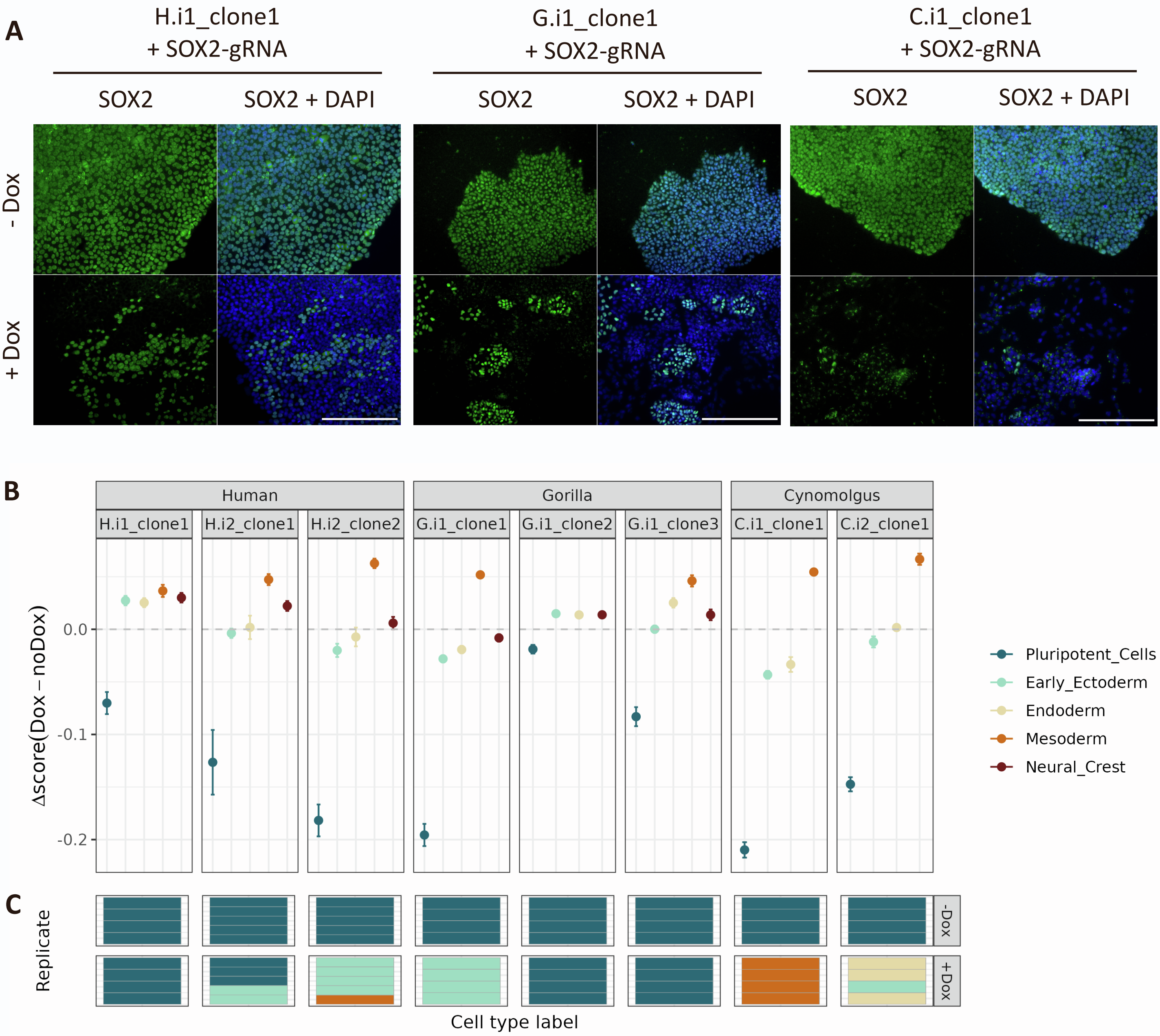

Figure S4. SOX2-knockdown and impact on cell fate. *Related to Figure 4.* (**A**) Immunofluorescence stainings for SOX2. KRAB-dCas9 iPSCs with an integrated SOX2-targeting gRNA were cultured in medium with or without 1 µg/mL dox for 4 days; scale bars indicate 250 µm. (**B**) Cell type classification with the RNA-seq data of cells with a SOX2-gRNA was performed using SingleR49 with reference data from Rhodes et al.[50] The Δ of the correlation scores for the cell types were determined between the score in the +dox and in the -dox condition with 0 indicating no change between the conditions (dotted horizontal line); data is represented as mean (n = 4-5 biological replicates) +/- SEM. (**C**) A cell type label was assigned by SingleR[49] to each biological replicate in the -dox and in the +dox condition indicating the highest correlation score of each sample to the reference. In the -dox condition all replicates from all clones showed the highest correlation to pluripotent cells, in the +dox condition the assigned cell type labels were more variable and in many cases the highest correlation score was now determined to differentiated cell types.

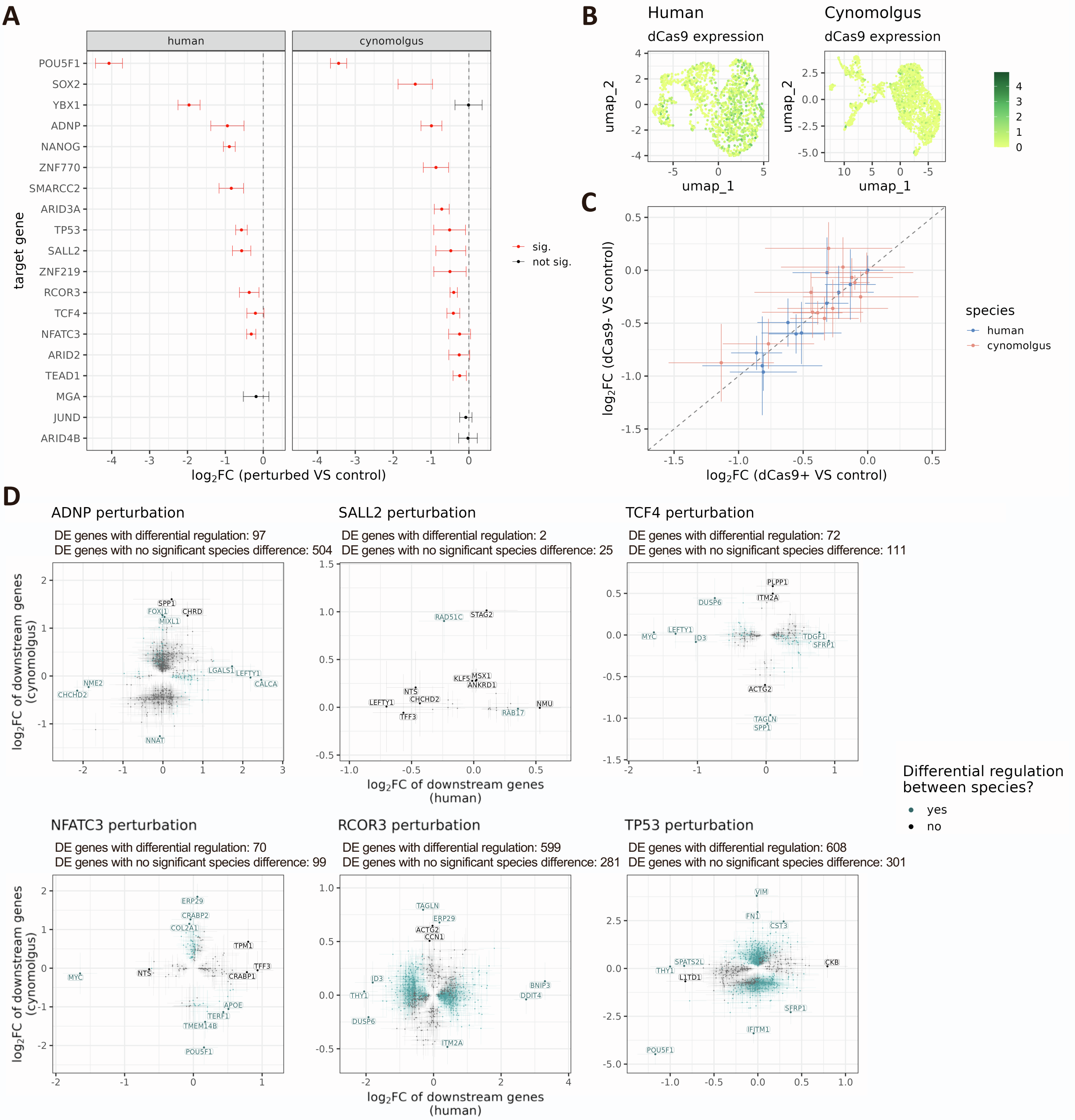

Figure S5. Single-cell CRISPR-screen. *Related to Figure 5.* (**A**) Log2 fold changes of the targeted genes in the CRISPR screen in perturbed VS control cells. Target genes that had a mean log-expression of ≥0.2 in both human and cynomolgus control cells and gRNAs that were detected in ≥10 perturbed cells are included. We performed the DE testing per gRNA using limma-trend and applied a significance threshold of adjusted p-value <0.1 (one-tailed test). Only the best performing gRNA per gene is displayed. (**B**) UMAP embeddings of the single-cell CRISPR screen data. Cells are colored by KRAB-dCas9 expression in logcounts. (**C**) Target genes are downregulated to a similar degree in cells where KRAB-dCas9 transcripts were detected (≥ 1 UMI counts) and in cells where KRAB-dCas9 was not detected. We analyzed target genes that had a mean log-expression of ≥0.2 in both human and cynomolgus control cells and gRNAs that were detected in ≥10 dCas9+ and ≥10 dCas9- cells. Each data point depicts the effect of a specific gRNA in a specific species (denoted by the color). Based on the similar effects we observed here and the findings in Figure 5E, we kept cells defined as dCas9- for further analysis. (**D**) Downstream transcriptomic effects of the perturbed target genes that were significantly downregulated and fulfilled minimal detection criteria in both species (for POU5F1 downstream effects see Figure 5G). The log2 fold changes (perturbed VS control) in cynomolgus are plotted against the log2 fold changes in human per perturbation for the genes that are significantly differentially expressed in at least one species. Data points are colored by differential regulation between species (significance threshold for differential expression and differential regulation: adj. p-value <0.05). The number of data points for each color is indicated in the subtitles. The top 5 genes with the highest significant log2 fold change for each contrast - perturbed VS control in human, perturbed VS control in cynomolgus and species:condition interaction term, i.e. differential regulation - are labeled per perturbation (overlaps are possible).

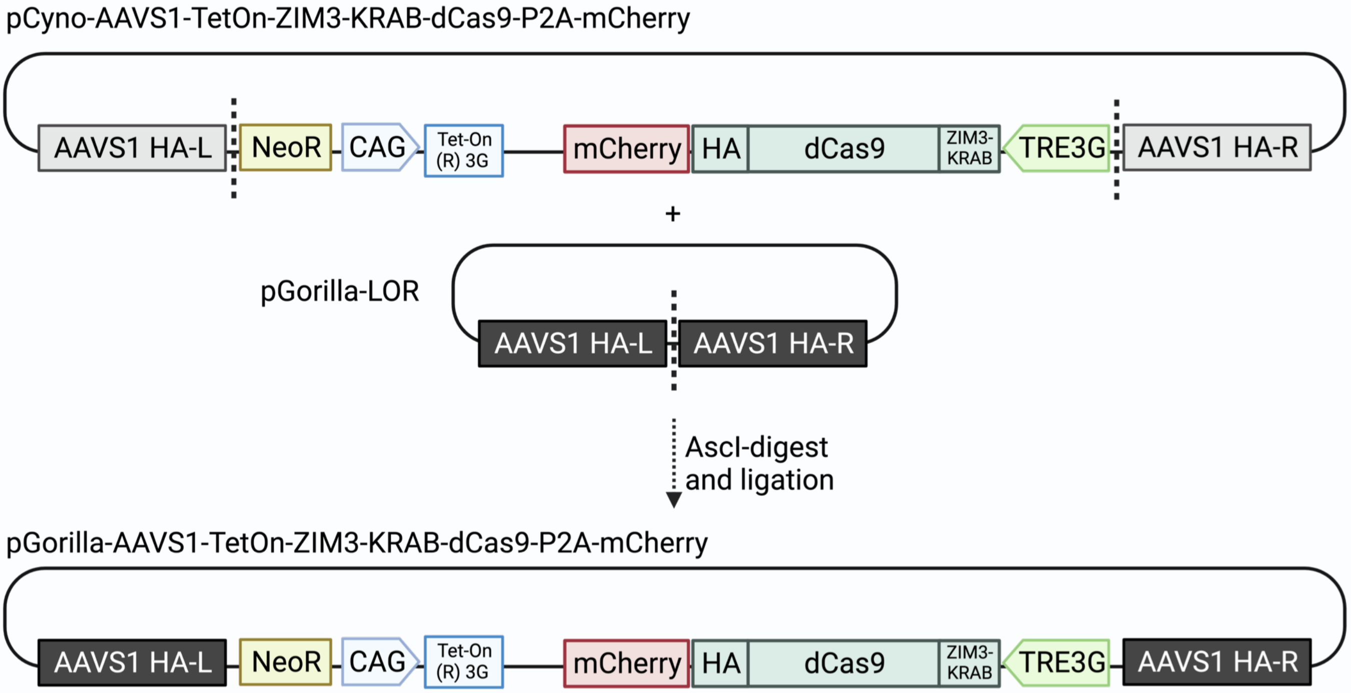

Figure S6. Species-specific pAAVS1 cloning scheme. *Related to STAR Methods.* To target the AAVS1-locus of different species, the species-specific AAVS1 homology arms of the KRAB-dCas9-cassette-coding donor plasmid can be exchanged. By one cut and ligation step the donor plasmid and a plasmid that encodes homology arms to the AAVS1 locus of another species can be combined. Like that we generated our the final pGorilla-AAVS1-TetOn-ZIM3-KRAB-dCas9-P2A-mCherry, by combining the cynomolgus donor plasmid pCyno-AAVS1-TetOn-ZIM3-KRAB-dCas9-P2A-mCherry and the pGorilla-LOR. Briefly, 1 ng of the cynomolgus donor plasmid (pCyno-AAVS1-TetOn-ZIM3-KRAB-dCas9-P2A-mCherry) and 1 ng of the species-specific pLOR coding the AAVS1-targeting homology arm (here pGorilla-LOR) were AscI-digested, gel-purified and ligated in a 1:5 ratio with a T4 ligase. This resulted in the final gorilla donor plasmid, pGorilla-AAVS1-TetOn-ZIM3-KRAB-dCas9-P2A-mCherry; (AAVS1 HA-L = AAVS1 homology arm left, AAVS1 HA-R = AAVS1 homology arm right).
